# PRC1‐Mediated H2Aub Loop Formation and Function in *Arabidopsis*

**DOI:** 10.1002/advs.202504377

**Published:** 2025-07-12

**Authors:** Lingxiao Luo, Minqi Yang, Zhihan Song, Yali Liu, Suxin Xiao, Dingyue Wang, Myriam Calonje, Hang He, Yue Zhou

**Affiliations:** ^1^ State Key Laboratory of Gene Function and Modulation Research School of Advanced Agricultural Sciences Peking‐Tsinghua Center for Life Sciences Peking University Beijing 100871 China; ^2^ Institute of Cell Biology and MOE Key Laboratory of Cell Activities and Stress Adaptations School of Life Sciences Lanzhou University Lanzhou 730000 China; ^3^ Institute of Plant Biochemistry and Photosynthesis (IBVF‐CSIC) Avenida Américo Vespucio 49 Seville 41092 Spain

**Keywords:** BMI1s, chromatin loops, gene repression, H2Aub, PRC1, RING1s

## Abstract

Polycomb group (PcG) complexes play a crucial role in the regulation of gene repression and in organizing the 3D conformation of chromatin. However, while Polycomb Repressive Complex (PRC) 2 mediated H3K27me3 clearly contributes to the formation of repressive chromatin loops in *Arabidopsis*, the role of PRC1 mediated H2Aub remains unclear. Notably, in the *Arabidopsis* genome, H2Aub and H3K27me3 marks do not always co‐localize, and some regions are marked exclusively by H2Aub, but the contribution of these only‐H2Aub regions to chromatin looping remains largely unexplored. By using Capture‐High‐throughput Chromosome Conformation Capture (C‐Hi‐C) to specifically enrich interactions associated with H2Aub regions, it is find that H2Aub loops are preferentially established between two H2Aub enriched regions. Although pure only‐H2Aub‐loops can be detected, H2Aub regions tend to interact with regions marked by both H2Aub and H3K27me3, contributing to the formation of PcG hubs. Furthermore, it is find that maintaining appropriate level of H2Aub and H3K27me3 is essential for the stability of H2Aub loops and that the formation of these loops reinforce gene repression. This findings unveil the existence of H2Aub loops in *Arabidopsis* and their impact on transcriptional regulation.

## Graphical abstract

In the *Arabidopsis* genome, H2Aub and H3K27me3 marks do not always co‐localize, and some regions are marked exclusively by H2Aub. By using Capture‐Hi‐C, it is found that H2Aub loops are preferentially established between two H2Aub enriched regions. H2Aub regions tend to interact with regions marked by both H2Aub and H3K27me3, facilitating PcG hub assembly for transcriptional repression.

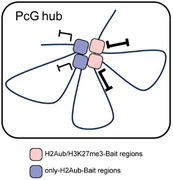

## 1 Introduction

Polycomb group (PcG) proteins form two major complexes, Polycomb Repressive Complex (PRC) 1 and PRC2, which modify the chromatin by incorporating H2Aub and H3K27me3, respectively. These modifications play a crucial role in chromatin organization, thereby controlling the spatiotemporal expression of genes.^[^ ^1^ ^]^ PcG proteins were first identified in *Drosophila* and are evolutionary conserved across species.^[^ ^2^ ^]^ PRC1 exhibits greater compositional diversity than PRC2. PRC1 contains an H2A E3 monoubiquitin ligase module, which pairs with specific components to form distinct PRC1 complexes. The E3 module, together with the accessory proteins Polycomb (Pc), Polyhomeotic (Ph) and Sex Comb on Midleg (Scm) form the canonical PRC1 (cPRC1) complex. By contrast, variant PRC1 (vPRC1) complexes incorporate Ring1 and Yin Yang 1 binding protein (Rybp) as accessory proteins.^[^ ^1^, ^2^ ^]^ Feedback regulation between PRC1 and PRC2 stabilizes gene silencing. On the one hand, PRC2 initially establishes H3K27me3, which leads PRC1 to recognize through the subunit Pc and maintain stable repression through depositing H2Aub or other mechanisms.^[^ ^3^ ^]^ On the other hand, vPRC1 initially mediates H2Aub at its target, subsequently recruiting PRC2 for H3K27me3 marking.^[^ ^4^ ^]^

In *Arabidopsis*, REALLY INTERESTING NEW GENE 1A/B (RING1A/B) and B‐CELL‐SPECIFIC MOLONEY MURINE LEUKAEMIA VIRUS INTEGRATION SITE 1A/B/C (BMI1A/B/C) have been characterized as E3 module proteins. Unlike in *Drosophila*, where only RING1 proteins provide catalytic activity while some accessory proteins enhance enzymatic activity, in *Arabidopsis*, RING1A/B and BMI1A/B/C possess H2A ubiquitin ligase activity.^[^ ^5^ ^]^ No plant homologs of Pc, Ph, Rybp, or other accessory proteins have been identified.^[^ ^1^, ^6^ ^]^ Instead, two plant‐specific proteins, EMBRYONIC FLOWER 1 (EMF1) and LIKE‐HETEROCHROMATIN PROTEIN 1 (LHP1), have also been identified as PRC1 components.^[^ ^1a^ ^]^ EMF1 functions as chromatin organizer and LHP1 is the reader of H3K27me3.^[^ ^7^ ^]^ Unlike in animals, where both cPRC1 and vPRC1 exist, *Arabidopsis* appears to lack a cPRC1. Evidence suggests that H2Aub deposition is independent of PRC2 activity, as a reduction in H3K27me3 but not H2Aub was observed in the PRC2 mutant *clfswn*. Conversely, the loss of the E3 ligases BMI1A/B/C or RING1A/B leads to decreased levels of both H2Aub and H3K27me3 at loci where the two marks co‐localize.^[^ ^8^ ^]^ Moreover, in contrast to the extensive co‐localization of H2Aub and H3K27me3 in animals, these marks do not overlap as extensively in the *Arabidopsis* genome. Instead, PcG targets can be classified into three distinct subsets: those marked only by H2Aub, only by H3K27me3, or by both modifications. Transcriptional analyses suggest that these two histone modifications do not have identical roles in gene regulation.^[^ ^8^ ^]^ However, the mechanisms underlying the formation of these subsets, their regulation, and their interrelationships is not fully understood.

In interphase, the chromatin is hierarchically folded in the nucleus, forming four well‐established structures: chromatin loops, topologically associated domain (TAD), A/B compartment, and chromosome territories (CTs).^[^ ^9^ ^]^ PcG proteins are involved in 3D chromatin organization and have been reported to form chromatin loops by facilitating interactions between two distinct genomic regions, which influence gene transcription.^[^ ^1b^ ^]^ Nevertheless, while PRC2/H3K27me3 interactions play a clear role in transcription repression, the influence of PRC1/H2Aub interactions on gene transcription is not as straightforward. For instance, it has been reported that PRC1 physically constrains developmental transcription factor genes and their enhancers in a silenced but poised spatial network in mouse embryonic stem cells (mESCs),^[^ ^10^ ^]^ while other results indicate that RING1 mediates the interaction of the promoter with a tissue‐specific enhancer to allow the activation of *Meis2* in mouse midbrain.^[^ ^11^ ^]^ In addition, it has been shown that in *Drosophila* eye‐antennal imaginal discs (EDs) and mouse neural progenitor stem cells (NPCs), PRC1 components bind to a subset of active regulatory elements and contribute to the formation of enhancer‐promoter loops, mediating the gene activation.^[^ ^12^ ^]^ Thus, PRC1/H2Aub interactions seem have a more versatile role.

Similar to *Drosophila* and mammals, PRC2/H3K27me3 has been reported to play a key role in the formation and maintenance of repressive chromatin loops in plants. Several studies have shown that H3K27me3‐enriched regions tend to physically interact, and that PRC2/H3K27me3 is essential for maintaining these chromatin loops not only in *Arabidopsis*,^[^ ^13^ ^]^ but also in crops such as soybean and rice.^[^ ^13^, ^14^ ^]^ As for PRC1/H2Aub, a recent study in *Arabidopsis* revealed its involvement in various types of interactions. First, PRC1 cooperates with PRC2/H3K27me3 to maintain the interactions within TADs or compartment domains (CDs). Second, it hinders the formation of gene loops. Third, it prevents aberrant long‐range interactions of H3K27me1 regions.^[^ ^15^ ^]^ Previous research focused on the influence of H2Aub on multiple types of chromatin loops, yet it remains unclear whether PRC1‐mediated H2Aub loops exist. The spatial relationship between PRC1/H2Aub and PRC2/H3K27me3‐enriched regions is also unknown.

Here, we performed Capture‐High‐throughput Chromosome Conformation Capture (C‐Hi‐C) to specifically enrich interactions associated with H2Aub‐enriched regions (H2Aub loops), providing genome‐wide insights into PRC1‐mediated H2Aub looping. By integrating these data with Chromatin Immunoprecipitation Sequencing (ChIP‐Seq) results, we found that H2Aub‐enriched regions preferentially interact with other regions marked by the same histone modification. Furthermore, despite the existence of three distinct types of PcG targets in *Arabidopsis*, only‐H2Aub regions do not segregate from H3K27me3‐marked regions in 3D nuclear space. Instead, they tend to cluster with regions marked by both H2Aub and H3K27me3, forming PcG hubs. C‐Hi‐C analyses in the PRC1 mutants *bmi1a/b/c* and *ring1a/b*, revealed that appropriate histone modification levels are crucial for maintaining H2Aub loops. Moreover, H2Aub loop formation reinforces PcG‐mediated repression. Interestingly, the loss of PcG‐deposited histone modifications in *bmi1a/b/c* and *ring1a/b* differentially disrupts chromatin loop organization. These findings unveil the spatial relationships of H2Aub‐enriched regions and their physical interactions, shedding light on the role of PRC1/H2Aub in chromatin looping and transcriptional regulation.

## 2 Results

### 2.1 H2Aub‐Enriched Regions Tend to Interact with Regions Enriched with the Same Histone Modification

**Figure 1:**
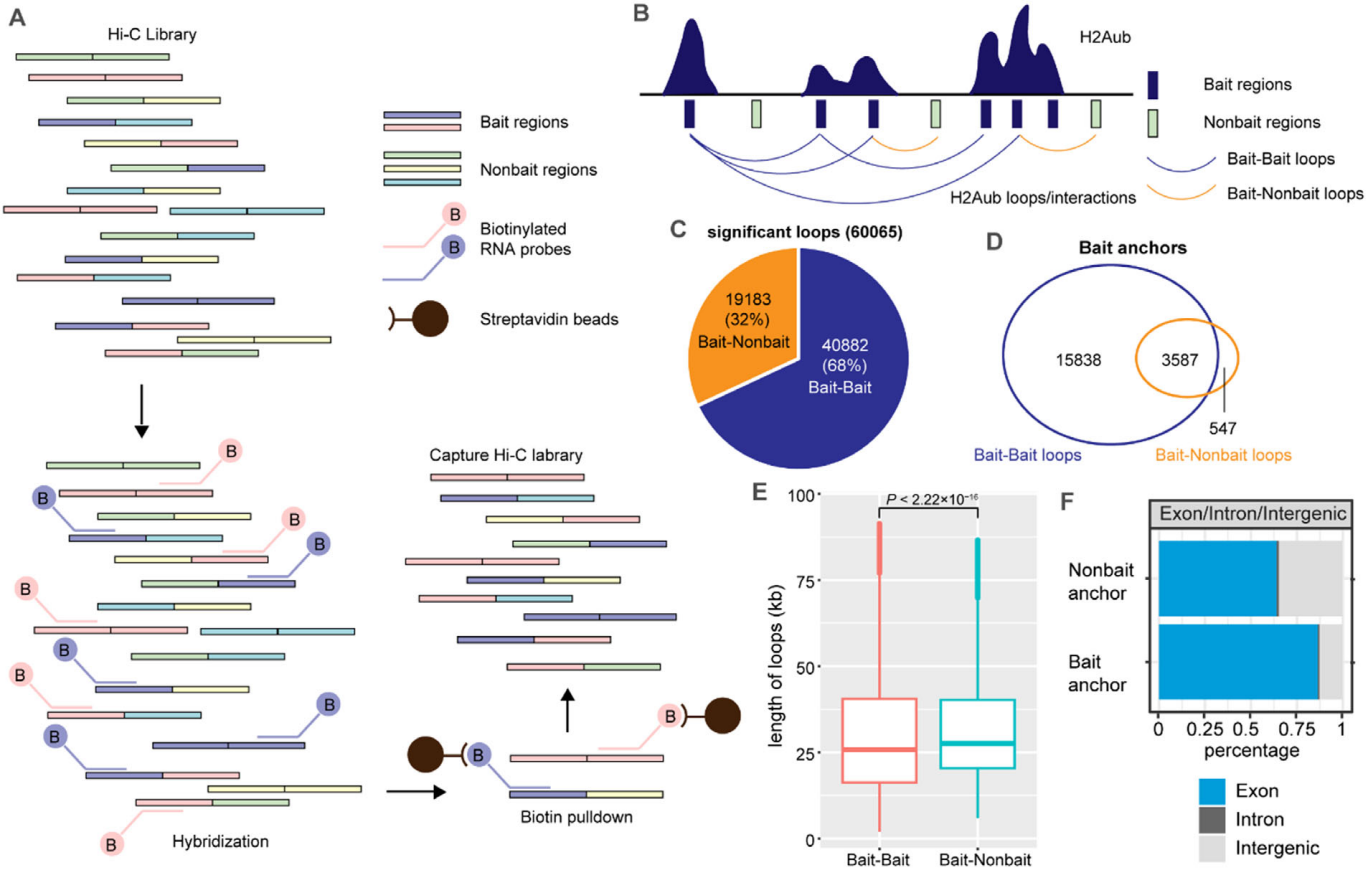
H2Aub‐enriched regions tend to interact with regions enriched with the same histone modification. A) Schematic representation of Capture Hi‐C. Hi‐C libraries containing all interactions are first generated. The RNA bait library, labeled with biotin, is artificially synthesized and hybridized with the Hi‐C library. Biotin‐labeled fragments are pulled down using Streptavidin beads and then amplified by PCR, enriching the targeted interactions and generating the corresponding C‐Hi‐C library. B) Schematic diagram showing the spatial interaction or separation of different genomic regions. Region pairs that interact in space serve as anchors of a loop. Bait regions are H2Aub‐enriched regions and serve as the binding sites of RNA probes or the *Dpn* II restriction fragments. H2Aub loops are formed by interactions between two anchors, with at least one being a Bait region. Bait‐bait loops are formed by the interactions between two Bait regions, whereas Bait‐Nonbait loops are formed by the interactions between one Bait region and one Nonbait region. C) Pie plot showing the amount and proportional situation of Bait‐Bait and Bait‐Nonbait loops among all H2Aub loops. D) Venn diagram showing the overlap of Bait anchors between Bait‐Bait loops and Bait‐Nonbait loops. Anchors are *Dpn* II restriction fragments that can interact with other genomic regions to form H2Aub loops. Bait anchors are those hybridized by RNA probes. After loop calling and classification, Bait anchors from Bait‐Bait loops and Bait‐Nonbait loops are identified. If two loops from different categories share a Bait anchor, it is defined as an overlapped Bait anchor. E) Box plots showing the lengths of Bait‐Bait and Bait‐Nonbait loops. The median (middle line) and upper and lower quartiles (boxes) are displayed. *P*‐values are calculated using the Wilcoxon rank‐sum test. F) Distribution of Nonbait (top) and Bait (bottom) anchors relative to exon or intron features.

To explore the influence of PRC1/H2Aub on chromatin loops, we performed C‐Hi‐C to enrich for interactions related with H2Aub enriched regions (H2Aub interactions) (**Figure** **1**A). Briefly, according to the H2Aub ChIP‐Seq results, a biotinylated RNA bait library specifically targeting 38 969 H2Aub Bait regions were generated, which almost covers all H2Aub enriched regions (Figure 1A,B; Figure S1A, Supporting Information). There is more than one probe matched to a single gene, and genes with H2Aub and H3K27me3 tend to have more probes associated with them compared to those only marked by H2Aub (Figure S1B, Supporting Information). We then conducted in situ Hi‐C experiments in wild type (WT)PATHOGENESIS‐RELATED GENE 5 seedlings and the libraries were hybridized with the designed RNA bait library to captured H2Aub specific interactions (Figure S2, Supporting Information). The captured libraries were then sequenced and the correlation analysis confirms the high reproducibility of the two replicates (Figure S3A–G, Supporting Information). Therefore, H2Aub interactions were efficiently captured using H2Aub C‐Hi‐C in *Arabidopsis*, which allowed for an in‐depth characterization of the genomic regions interacting with H2Aub‐enriched regions (Figure S3H, Supporting Information).

We identified 60065 significant H2Aub loop interactions. Considering the distribution of probes on the genes, there was also more than one loop connected with a single gene. The loops whose anchors located on the same genes still linked different interaction regions (Figure S4A, Supporting Information). The interaction regions of genes only marked by H2Aub were more concentrated than the genes with H2Aub and H3K27me3 (Figure S4B, Supporting Information). We further subdivided these H2Aub loop in two types. The first type refers to loops in which only one of the two anchors is an H2Aub Bait region (Bait‐Nonbait loop), and the second type corresponds to loops in which the two anchors are H2Aub Bait regions (Bait‐Bait loops) (Figure 1B). 68% of the loops were Bait‐Bait loops, while the remaining 32% were Bait‐Nonbait loops (Figure 1C). Examining the Bait anchors of these loops, as one Bait anchor can interact with more than one region, we found that most of the Bait anchors forming Bait‐Nonbait loops (3587) also established Bait‐Bait loops, indicating that there are very few Bait anchors (547) interacting only with Nonbait regions (Figure 1D). These data suggest that H2Aub enriched regions tend to interact with regions enriched in the same modification, rather than regions without H2Aub. The median length of Bait‐Bait loops is ≈25 kb, which is slightly shorter than that of Bait‐Nonbait loops (Figure 1E). Analyzing the genomic characteristics of Bait and Nonbait anchors, we found that the two types of anchors mainly localize at exons. However, compared to Bait anchors, Nonbait anchors are more frequently found in intergenic regions (Figure 1F). Taken together, our findings reveal the presence of PRC1‐mediated H2Aub loops in *Arabidopsis*, and H2Aub‐enriched regions preferentially interact with other H2Aub‐enriched regions.

### 2.2 Only‐H2Aub Regions Interact with H2Aub/H3K27me3 Regions in Space

In *Arabidopsis*, PRC2 mediated H3K27me3 has been reported to contribute to loop formation and/or maintenance.^[^ ^13^, ^15^ ^]^ Genome‐wide localization studies of H2Aub and H3K27me3 in *Arabidopsis* have shown that, H2Aub can be enriched independently or co‐enriched with H3K27me3.^[^ ^8a^ ^]^ Nevertheless, the relationship between regions only marked with H2Aub (here after only‐H2Aub) and regions marked with H2Aub/H3K27me3 in space is unknown. To examine this, we classified the Bait regions into only‐H2Aub‐Bait and H2Aub/H3K27me3‐Bait regions, based on the absence or presence of H3K27me3, respectively (Figure S5, Supporting Information).

**Figure 2:**
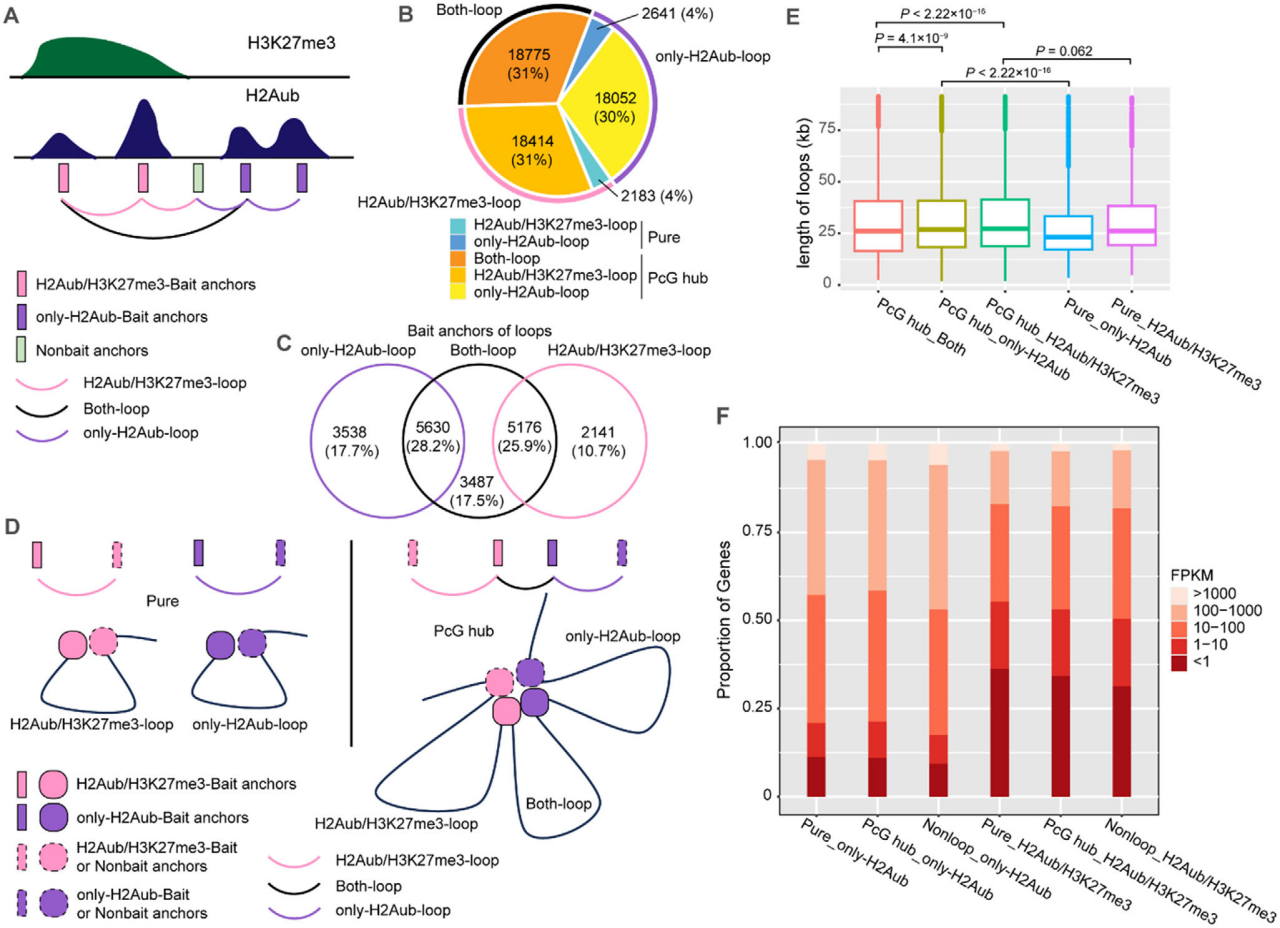
H2Aub‐enriched regions tend to form PcG hubs in space, and H2Aub loops repress transcription in concert with H3K27me3. A) Schematic diagram showing the classification of loop anchors and H2Aub loops. Anchors with or without H2Aub enrichment are Bait anchors and Nonbait anchors, respectively. Bait anchors further subdivided into only‐H2Aub‐Bait anchors and H2Aub/H3K27me3‐Bait anchors, based on the absence or presence of H3K27me3, respectively. Loops in which one anchor is only‐H2Aub region and the other is H2Aub/H3K27me3 region are Both‐loops (black). Loops in which both anchors lack H3K27me3 enrichment are defined as only‐H2Aub‐loops (purple). Loops in which at least one anchor is an H3K27me3‐enriched region and no anchor is only‐H2Aub‐Bait region are defined as H2Aub/H3K27me3‐loops (pink). B) Amount and proportions of different types of H2Aub loops. The outer ring shows the classifications mentioned in A). The inner ring shows the further classifications mentioned in D). C) Venn diagrams showing the amount and overlap of Bait anchors among Both‐loops, only‐H2Aub‐loops and H2Aub/H3K27me3‐loops. D) Schematic diagram showing the five types of H2Aub loops. Both‐loops are formed by interactions between an only‐H2Aub‐Bait region and an H2Aub/H3K27me3‐Bait region. Only‐H2Aub‐ and H2Aub/H3K27me3‐loops are formed by interactions between two only‐H2Aub‐Bait or H2Aub/H3K27me3‐Bait regions, respectively. Both‐loops share anchors with only‐H2Aub‐ or H2Aub/H3K27me3‐loops, clustering to form the PcG hub. The loops belonging to the PcG hub are further divided into PcG hub‐Both‐loops, PcG hub only‐H2Aub‐loops, and PcG hub H2Aub/H3K27me3‐loops. Only‐H2Aub‐ or H2Aub/H3K27me3‐loops, which are independent of PcG hub, are classified as Pure only‐H2Aub‐ and Pure H2Aub/H3K27me3‐loops, respectively. E) Box plots showing the lengths of PcG hub Both‐loops, PcG hub only‐H2Aub‐loops, PcG hub H2Aub/H3K27me3‐loops, Pure only‐H2Aub‐ and Pure H2Aub/H3K27me3‐loops (from left to right). The median (middle line) and upper and lower quartiles (boxes) are displayed. *P*‐values are calculated using the Wilcoxon rank‐sum test. F) Bar plots showing the proportional expression levels of genes. Expression levels of genes located at only‐H2Aub‐Bait regions or H2Aub/H3K27me3‐Bait regions belonging to Pure loops, PcG hub loops and Nonloop regions are compared.

First, we extracted all Both‐loops, defined as loops formed by interactions between only‐H2Aub‐Bait regions and H2Aub/H3K27me3‐Bait regions (**Figure** **2**A, black line). According to the principle of C‐Hi‐C, only loops containing at least one Bait anchor can be captured. Therefore, the remaining loops can be further subdivided into two categories based on the type of their Bait anchor, each containing only one type of Bait region. We defined loops with a Bait anchor in an only‐H2Aub‐Bait region as only‐H2Aub‐loops (Figure 2A, purple line) and those with a Bait anchor in an H2Aub/H3K27me3‐Bait region as H2Aub/H3K27me3‐loops (Figure 2A, pink line). Both‐loops accounted for 31% of all H2Aub loops, indicating that only‐H2Aub and H2Aub/H3H27me3 bait regions engage in spatial interactions (Figure 2B).

Interestingly, we further observed that other two types of loops also share the majority of bait anchors with the Both‐loops (Figure 2C). In other words, the majority of H2Aub‐related interactions participate in a hub started with the Both‐loop Bait anchors. We called this hub the PcG hub, and for the following statement, we defined the Both‐loops as PcG hub Both‐loops (Figure 2D). If only‐H2Aub‐loops shared anchors with Both‐loops, they are classified as PcG hub only‐H2Aub‐loops, indicating that they indirectly interact with H3K27me3‐enriched regions. Conversely, only‐H2Aub‐loops that are completely independent of H3K27me3 are categorized as Pure loops (Pure only‐H2Aub‐loops). Similarly, H2Aub/H3K27me3‐loops are subdivided into PcG hub H2Aub/H3K27me3‐loops and Pure H2Aub/H3K27me3‐loops based on the structure to which they belong (Figure 2D). We then compared the amount of Pure only‐H2Aub‐ and H2Aub/H3K27me3‐loops, and PcG hub only‐H2Aub‐, H2Aub/H3K27me3‐ and Both‐loops, finding that more than 90% of the loops belong to the PcG hub while only a few are Pure loops (Figure 2B). We also found that the length of Pure loops was shorter than that of loops within PcG hub, in particular that of Pure only‐H2Aub‐loops. For loops in the PcG hub, Both‐loops are the shortest (Figure 2E). In any case, these results indicate that, even in the absence of enrichment of H3K27me3 in only‐H2Aub‐Bait regions, these regions physically interact with H2Aub/H3K27me3 regions in space.

### 2.3 H2Aub Loops Repress Transcription in Concert with H3K27me3

We next explored whether H2Aub loop formation influences gene transcription. From the perspective of Bait regions, not all Bait regions interact with another genomic region to form a chromatin loop. Approximately 51% of Bait regions serve as loop anchors, with H2Aub/H3K27me3‐Bait regions (56%) being more likely to form loops than only‐H2Aub‐Bait regions (48%) (Figure S6, Supporting Information). To further examine the transcriptional impact of these chromatin structures, we divided Bait regions into six categories: forming PcG hub loop (PcG hub_only‐H2Aub, PcG hub_H2Aub/H3K27me3), forming Pure loop (Pure_only‐H2Aub, Pure_H2Aub/H3K27me3) and no forming loop (Nonloop_only‐H2Aub, Nonloop_H2Aub/H3K27me3). We then compared gene expression levels across these groups. Our findings reveal three important patterns. First, H2Aub/H3K27me3‐Bait regions exhibit lower expression levels than only‐H2Aub‐Bait regions, regardless of whether they are PcG hub, pure loop anchors, or Nonloop regions, confirming that H3K27me3 represses gene expression. Second, genes located at Bait anchors exhibit lower expression than those in Nonloop Bait regions, in the case of consistent histone modification at the regions, suggesting that H2Aub loop formation itself contributes to transcriptional repression. Third, genes at only‐H2Aub‐loop anchors are less transcriptionally active within PcG hubs than in Pure loops. By contrast, genes at H2Aub/H3K27me3‐loop anchors are more active within PcG hubs, indicating that greater spatial interactions with H3K27me3‐enriched regions correlate with lower gene expression at a Bait region (Figure 2F). These findings demonstrate that spatial interactions influence gene expression at Bait regions and that H2Aub loops repress transcription in concert with H3K27me3 to some extent.

Motif enrichment analysis showed that only‐H2Aub‐Bait anchors, irrespective of whether they form PcG hub or Pure loops, are enriched in CTCTGYTY motif. This motif is the binding site of RELATIVE OF EARLY FLOWERING 6 (REF6), which is an H3K27me3 demethylase in *Arabidopsis* that regulate the levels of this modification.^[^ ^16^ ^]^ In contrast, H2Aub/H3K27me3‐Bait anchors of PcG hub are enriched in RY elements recognized by B3 transcription factors (TFs),^[^ ^17^ ^]^ and Nonloop Bait regions in motifs recognized by GATA TFs (Figure S7, Supporting Information). These results suggest a possible implication of different factors in influencing loop formation.

### 2.4 The Maintenance of H2Aub Loops Requires an Appropriate Histone Modification Level at Anchors

**Figure 3:**
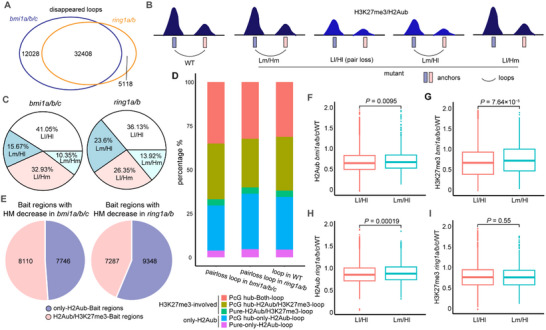
The maintenance of H2Aub loops requires an appropriate histone modification level at anchors. A) Venn diagram showing the overlap of disappeared loops in *bmi1a/b/c* and *ring1a/b* mutants. B) Schematic diagram illustrating the classification of loops in WT, based on whether a loop disappears and whether modification levels decrease at loop anchors in the mutant. “L” and “H” represent loop and histone modification levels, respectively. “l” and “m” indicate loss and maintenance of loops or histone modification, respectively. For example, Ll/Hl loops disappear in the mutant and are accompanied by histone modification loss at anchors. C) Proportions of the four loop types (Ll/Hl, Lm/Hl, Ll/Hm, Lm/Hm) in *bmi1a/b/c* (left) or *ring1a/b* (right) mutants. D) Bar plots showing the percentage of five types of loops that belong to pair loss loops in *bmi1a/b/c* and *ring1a/b*, or the loops in WT. E) Pie charts displaying the composition of Bait regions with decreased histone modification (HM, H2Aub or H3K27me3) levels in *bmi1a/b/c* (left) and *ring1a/b* (right). F–I) Box plots showing the degree of histone modification changes (mutant/WT) at unique bait anchors of Ll/Hl and Lm/Hl loops. The median (middle line) and upper and lower quartiles (boxes) are displayed. H2Aub and H3K27me3 level changes in *bmi1a/b/c* are shown in (F) and (G), respectively. Changes in the levels of H2Aub and H3K27me3 in *ring1a/b* are shown in (H) and (I), respectively. *P*‐values are calculated using the Wilcoxon rank‐sum test.

BMI1 and RING1 proteins are all E3 ligases and core components of PRC1 in *Arabidopsis*. Removal of either BMI1s or RING1s leads to the decrease of H2Aub and causes the loss of H3K27me3 at H2Aub/H3K27me3 marked regions.^[^ ^8^ ^]^ Therefore, to examine the influence of losing H2Aub or H3K27me3 histone modifications on H2Aub loops, we conducted H2Aub C‐Hi‐C in *bmi1a/b/c* and *ring1a/b* mutants (Figure S2, Supporting Information). We found that ≈40 000 H2Aub loops disappeared in both *bmi1a/b/c* and *ring1a/b* mutants compared to WT (**Figure** **3**A), supporting the importance of these histone modifications for loop formation and/or maintenance. We found a high number of loops disappearing in the two mutants, despite the fact that some loops disappear in only one of the mutants (Figure 3A). We then determined the percentage of loops (L) that are maintained (m) or disappeared (l) in relation to the maintenance (m) or loss (l) of histone modification (H, H2Aub or H3K27me3) at the anchors, thus, considering four categories: Ll/Hl (pair loss), Lm/Hm, Lm/HI, and Ll/Hm (Figure 3B). We found that the pair loss loops (Ll/Hl) represented the higher percentage in both *bmi1a/b/c* and *ring1a/b* mutants (Figure 3C). This result indicates a strong correlation between the loss of histone modifications and the loss of H2Aub loops in both mutants (Chi‐squared test, *P*‐value < 2.2 × 10^−16^).

Additionally, the loops containing at least one H2Aub/H3K27me3 Bait anchor (H3K27me3‐involved‐loops) are more affected in *bmi1a/b/c* (Chi‐squared test, *P*‐value < 2.2 × 10^−16^), whereas only‐H2Aub‐loops are more affected in *ring1a/b* (Chi‐squared test, *P*‐value = 1.449 × 10^−6^) (Figure 3D). This further supports the correlation between loop loss and histone modification loss since more H2Aub/H3K27me3 Bait regions have decreased histone modifications level in *bmi1a/b/c*, while more only‐H2Aub‐Bait regions lose histone modifications in *ring1a/b* (Figure 3E). Although BMI1s and RING1s are all E3 ligases in PRC1, they may have independent roles in regulating the histone modification and the corresponding H2Aub loop formation. In line with this, in *bmi1a/b/c* mutant, the decline of both H2Aub and H3K27me3 at Bait anchors are pronounced at loops that disappeared (Ll/Hl) than at those maintained (Lm/Hl) (Figure 3F,G), whereas in *ring1a/b* only a significant reduction of H2Aub was observed at Ll/Hl compared to Lm/HI (Figure 3H,I). Taken together, these data support that loops with more dramatic histone modification loss at their anchors are more likely to disappear, and maintenance of H2Aub loops requires appropriate histone modification levels at anchors.

### 2.5 The Combined Loss of H2Aub Loops and Histone Modifications Exacerbates Abnormal Gene Activation

**Figure 4:**
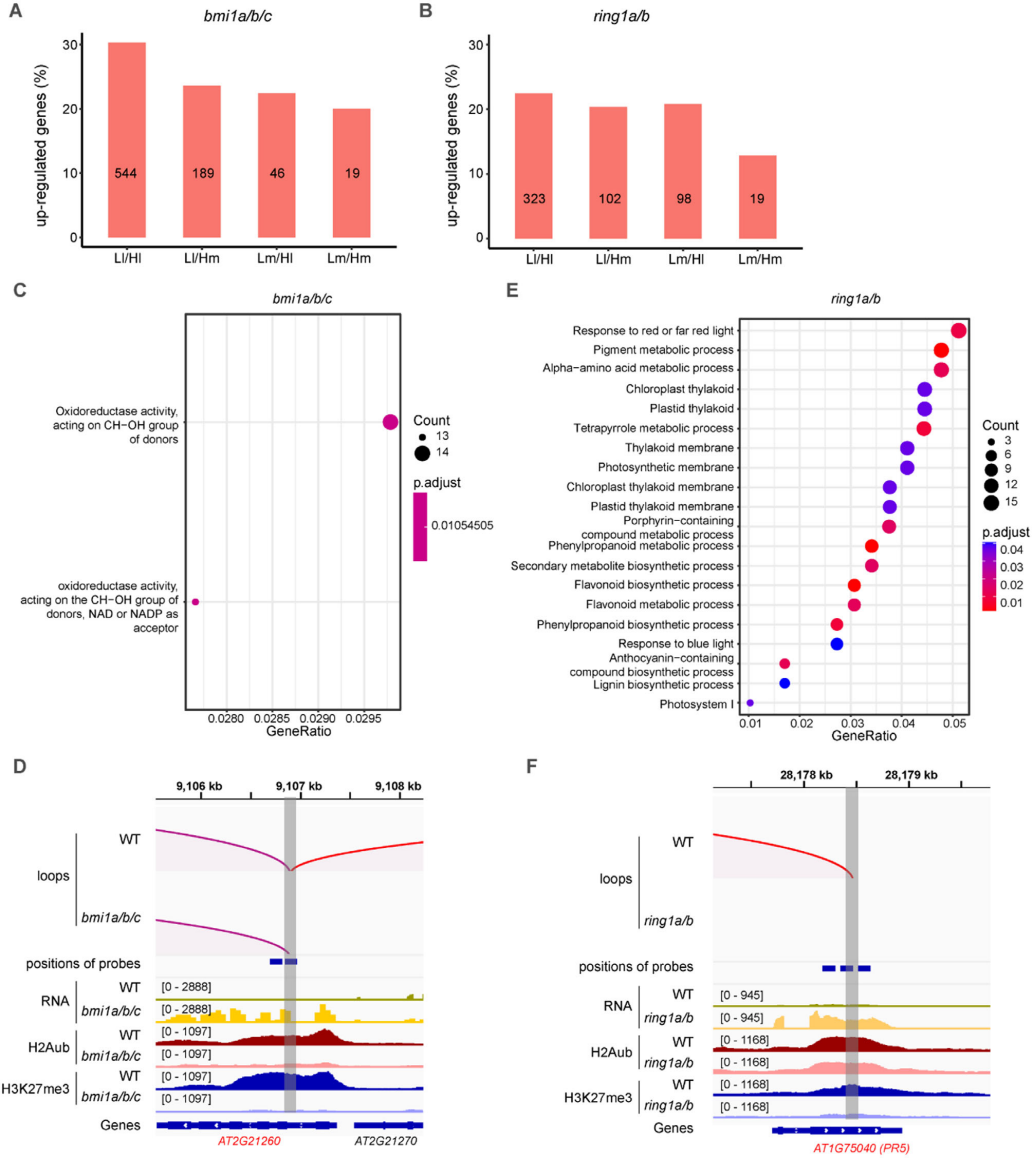
The combined loss of H2Aub loops and histone modifications exacerbates abnormal gene activation. A,B) Bar plots showing the percentage of up‐regulated genes in *bmi1a/b/c* (A) and *ring1a/b* (B). The results are displayed at the anchors belonging to four types of the loop, which are Ll/Hl, Ll/Hm, Lm/Hl, and Lm/Hm loops from left to right. Gene counts in each group are shown within the bars. C,E) Gene ontology (GO) enrichment analysis of up‐regulated genes at pair loss (Ll/Hl, loops disappear in the mutant and are accompanied by histone modification loss at anchors) loop anchors in *bmi1a/b/c* (C) and *ring1a/b* (E). Dot size represents the gene count, and the color gradient indicates the adjusted *P*‐value (p.adjust). D,F) Examples of up‐regulated genes located at Ll/Hl loops anchors in *bmi1a/b/c* (D) and *ring1a/b* (F). H2Aub loops, the position of probes, gene expression level, H2Aub level and H3K27me3 level, gene regions in WT plants, *bmi1a/b/c*, and *ring1a/b* mutants are presented. Representative up‐regulated genes are highlighted in red. Loops for which one anchor overlaps with the example gene are shown. H2Aub, H3K27me3 and RNA data are presented in reads per kilobase per million mapped reads (RPKM). The loop validated by 3C‐qPCR is indicated in red. The shaded region marks the location of the Ll/Hl loop anchor used as the fixed point in the 3C‐qPCR assay (Figure S11A,C, Supporting Information).

Given that the loss of H2Aub loops is often accompanied by histone modification loss at anchor regions in the two E3 ligase mutants, we next investigated how the loss of loop structure and histone modifications affect gene expression at loop anchors (**Figure** **4**; Figures S8,S9, Supporting Information). First, we found that the loss of H2Aub loops and decreased histone modification levels lead to the abnormal activation of genes at corresponding loop anchors in *bmi1a/b/c* mutant. Compared to anchors that retain loop interactions and histone modifications (Lm/Hm) in *bmi1a/b/c* mutant, anchors that lost only the interactions (Ll/Hm) or only histone modifications (Lm/Hl) exhibit a higher proportion of up‐regulated genes (Figure 4A). In addition, gene expression levels at anchors of Ll (disappeared loops) were significantly higher in *bmi1a/b/c* than in WT. By contrast, no noticeable changes in gene expression were observed for genes at anchors of Lm (maintained loops) (Figure S9A, Supporting Information). Second, the simultaneous loss of H2Aub loops and histone modifications further enhance abnormal gene activation, as the anchors that lost interactions and histone modifications (Ll/Hl) in *bmi1a/b/c* mutant show the highest proportion of up‐regulated genes (Figure 4A). This pattern is also observed in *ring1a/b* mutant (Figure 4B; Figure S9B, Supporting Information). The molecular changes in the two E3 ligase mutants collectively show that histone modifications deposited by PcG and the H2Aub loop structure contribute to transcriptional repression cooperatively.

Although many up‐regulated genes significantly overlapped between the *bmi1a/b/c* and *ring1a/b* mutants, each class of E3 ligases also independently regulated a substantial proportion of genes (Figure S10A, Supporting Information). This is consistent with the observation that, although BMI1s and RING1s are E3 ligases of PRC1, *bmi1a/b/c* and *ring1a/b* mutants exhibit different phenotypes (Figure S2, Supporting Information). A similar pattern was observed for up‐regulated genes associated with Ll/Hl loop anchors (Figure S10B, Supporting Information).

Gene Ontology (GO) analysis shows that the genes derepressed by both histone modifications and the loop structure in *bmi1a/b/c* are related to oxidoreductase activity, such as *AT2G21260* encoding an NAD(P)‐linked oxidoreductase superfamily protein (Figure 4C,D). The ectopic expression of oxidoreductase can cause oxidative damage, disrupt fundamental biological processes, including photosynthesis and respiration, and contribute to other cellular dysfunctions.^[^ ^18^ ^]^ This is consistent with the severe growth and development phenotype in *bmi1a/b/c* mutant (Figure S2, Supporting Information). By contrast, in *ring1a/b* mutant, genes enriched in GO terms are predominantly involved in light response, pigment synthesis, and metabolism (Figure 4E). The gene encoded PATHOGENESIS‐RELATED GENE 5 (PR5), which is participated in regulation of anthocyanin biosynthetic process and response to ultraviolet (UV) light,^[^ ^19^ ^]^ locates within Bait regions and forms H2Aub loops (Figure 4F). In *ring1a/b* mutant, same as *AT2G21260* in *bmi1a/b/c* mutant, the disappearance of specific H2Aub loops, accompanied by a significant decrease in H2Aub levels at Bait regions, leads to its abnormal activation. One representative loop and its associated changes in gene expression were further validated by 3C‐qPCR and RT‐qPCR, respectively. The loop located on the *AT2G21260* were lost in *bmi1abc* (Figure S11A, Supporting Information), and its expression was increased in the mutant (Figure S11B, Supporting Information). Similarly, the interaction on the *PR5* is also barely detected in 3C‐qPCR (Figure S11C, Supporting Information), with the higher expression in the *ring1ab* mutant (Figure S11D, Supporting Information). Altogether, collaborative loss of H2Aub loops and histone modifications in mutants enhances abnormal gene activation, disrupting the normal growth and metabolism of plants.

### 2.6 H2Aub Loops are Differentially Disturbed in *bmi1a/b/c* and *Ring1a/b* Mutants

**Figure 5:**
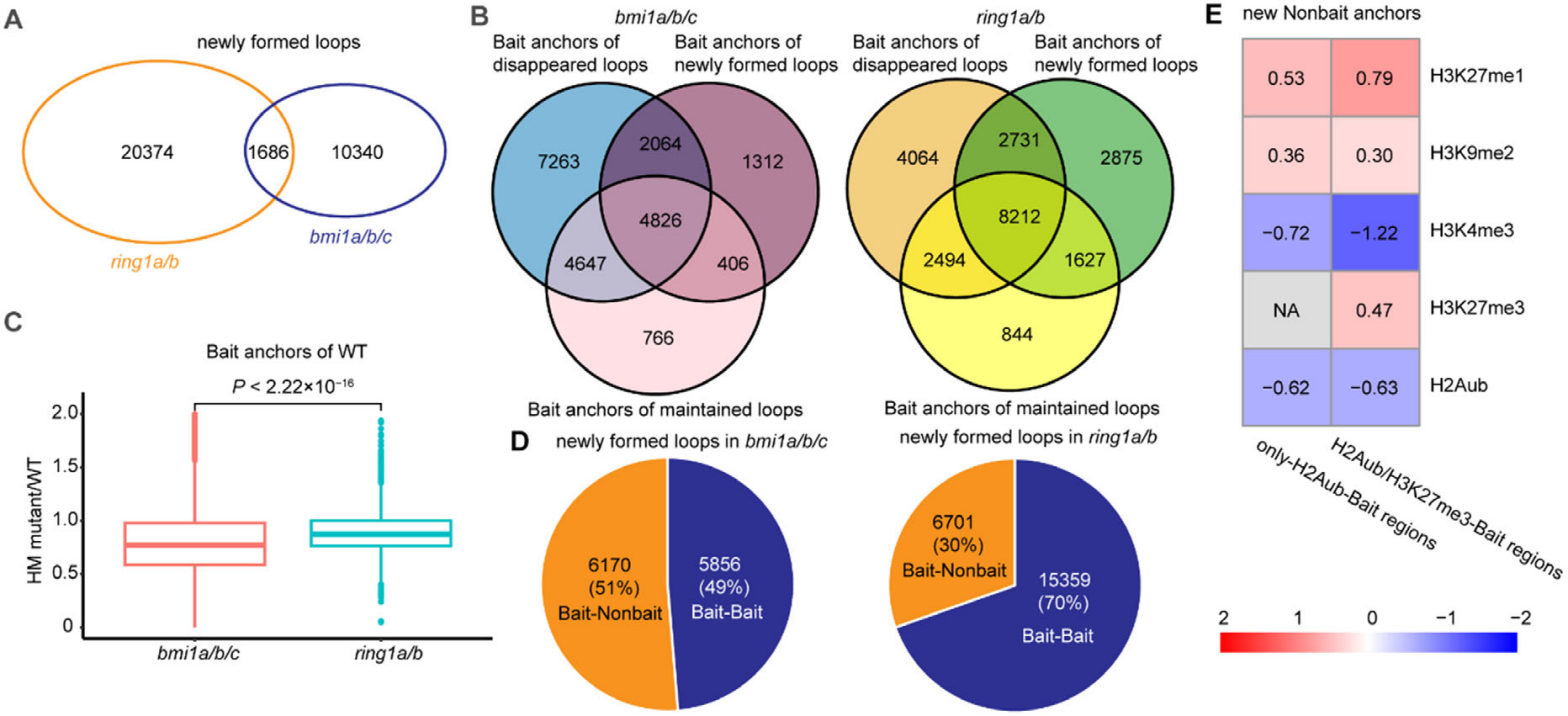
H2Aub loops are differentially disrupted in *bmi1a/b/c* and *ring1a/b* mutants. A) Venn diagram showing the overlap of the newly formed loops in *bmi1a/b/c* and *ring1a/b* mutants. B) Venn diagrams showing the overlap of bait anchors associated with disappeared loops, maintained loops, and the newly formed loops in *bmi1a/b/c* (left) and *ring1a/b* (right) mutants. Disappeared loops are the H2Aub loops present in WT but absent in the mutant. Maintained loops are the H2Aub loops that persist in both WT and the mutant. Newly formed loops are the H2Aub loops exclusively present in the mutant. C) Box plots showing the degree of histone modification (HM, H2Aub and H3K27me3) changes (mutant/WT) at WT bait anchors in *bmi1a/b/c*, and *ring1a/b* mutants. The median (middle line) and upper and lower quartiles (boxes) are displayed. *P*‐values are calculated using the Wilcoxon rank‐sum test. D) Proportions of Bait‐Bait and Bait‐Nonbait loops among newly formed H2Aub loops in *bmi1a/b/c* (left) and *ring1a/b* (right) mutants. E) Histone modification enrichment analysis at new Nonbait anchors in *bmi1a/b/c*. New Nonbait anchors are categorized based on their interaction with either only‐H2Aub‐Bait regions (left) or H2Aub/H3K27me3‐Bait regions (right). Pairwise Mann–Whitney U tests, corrected by the Bonferroni–Holm method for multiple comparisons, are used to determine the statistical significance of enrichment or depletion (target regions versus randomly selected regions). Enriched modifications are shown in red, and depleted modifications are in blue. Non‐significant modifications (*P*‐value > 0.05) are indicated in gray. Published data are obtained from the Plant Chromatin State Database and are listed in Table S2 (Supporting Information).

In addition to analyzing the changes in existing loops in WT upon the loss of BMI1s and RING1s, we also observed the formation of a comparable number of new H2Aub loops in *bmi1a/b/c* and *ring1a/b* mutants. Unlike the disappeared loops, these newly formed loops are different in the two mutants (**Figure** **5**A). These abnormal loops may result from recombination of the original anchors or involve new anchors that are nonloop regions in WT. Comparing the Bait anchors of disappeared, maintained, and newly formed loops, we found that these categories overlap to some extent in both mutants, confirming that the newly formed H2Aub loops are not entirely independent of normal loops in WT. However, in *bmi1a/b/c*, the number of WT Bait anchors that lost loop formation in the mutant is greater than other types of Bait anchors. By contrast, in *ring1a/b*, the number of Bait anchors associated with all three loop types is the highest (Figure 5B). Integrating these findings with results showing that more abnormal H2Aub loops form in *ring1a/b* than in *bmi1a/b/c* (Figure 5A), while more original H2Aub loops disappear in *bmi1a/b/c* than in *ring1a/b* (Figure 3A), we propose the following model: in *bmi1a/b/c*, normal H2Aub loops in WT are more thoroughly lost, than *ring1a/b* leading to a greater overall depletion of loops. In *ring1a/b*, more original Bait anchors shift their interactions, either exchanging interacting regions or interacting with nonloop regions of WT to form abnormal loops, resulting in overall loop disorganization. Furthermore, WT Bait anchors exhibit a more dramatic decrease in histone modification levels in *bmi1a/b/c* than in *ring1a/b* (Figure 5C), which may explain why fewer WT Bait anchors form new abnormal loops in *bmi1a/b/c*.

In WT, Bait‐Bait loops represented more than two‐thirds of the H2Aub loops (Figure 1C). A similar proportion was observed for the newly formed loops in *ring1a/b* (Figure 5D), indicating new interactions among WT Bait anchors. Conversely, the number of newly formed Bait‐Nonbait loops in *bmi1a/b/c* exceeded that of Bait‐Bait loops (Figure 5D), indicating that more Nonbait regions participate in new loop formation in this mutant. To explore the characteristics of these new Nonbait anchors in *bmi1a/b/c*, we analyzed the levels of different histone modifications at these regions in WT. We found that the new Nonbait anchors were enriched with H3K27me1 and H3K9me2, regardless of whether they interacted with only‐H2Aub‐Bait or H2Aub/H3K27me3‐Bait anchors (Figure 5E). Since H3K27me1 and H3K9me2 are repressive marks mainly associated with heterochromatin, these results suggest that normal H2Aub loops might hinder abnormal interaction between H2Aub enriched regions and heterochromatin regions. In addition, we found that H2Aub/H3K27me3‐Bait regions interacted with new Nonbait anchors enriched in H3K27me3, suggesting that normal H2Aub loops prevent the interaction between H2Aub/H3K27me3 Bait regions and other only‐H3K27me3 enriched regions (Figures 5E).^[^ ^8^, ^13^ ^]^ Accordingly, Nonbait anchors of H2Aub loops in WT were not enriched with H3K27me3 (Figure S12, Supporting Information). Based on these characteristics in the *bmi1a/b/c* mutant, genes at new anchors (anchors associated only with newly formed loops in the mutant) were more down‐regulated than those in *ring1a/b* mutant (Figure S13, Supporting Information), suggesting that normal H2Aub loops may prevent the excessive gathering of H3K27me3 enriched regions and provide a structure for fine tuning the gene expression.

## 3 Discussion

**Figure 6:**
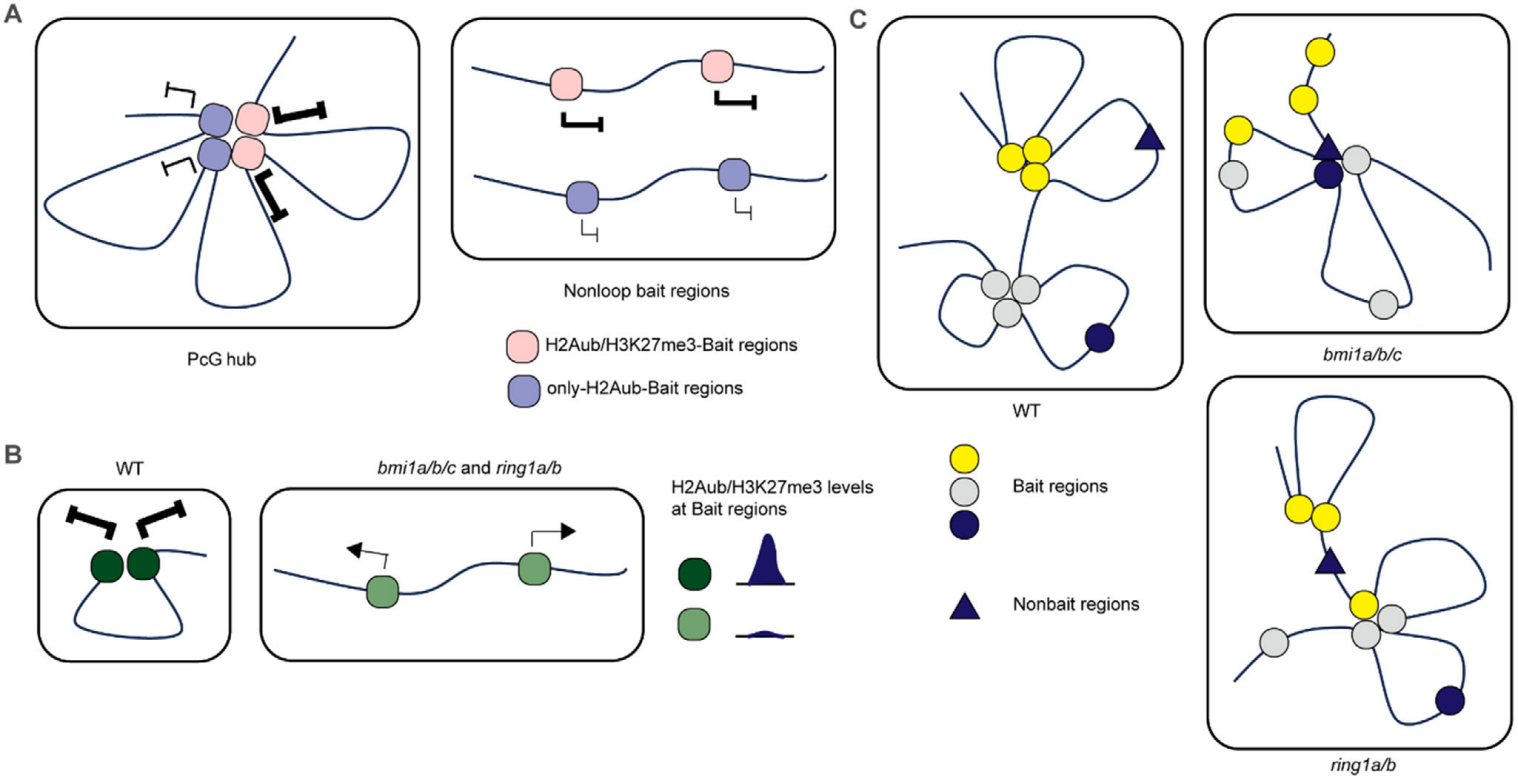
The working model for formation and function of PRC1‐mediated H2Aub loops. A) In WT plants, only‐H2Aub regions interact with H2Aub/H3K27me3 marked regions in space. H2Aub loops contribute to gene repression. B) In *bmi1a/b/c* and *ring1a/b* mutants, H2Aub loops tend to disappear, accompanied by the loss of histone modifications at loop anchors. The collaborative loss of H2Aub loops and histone modifications enhances abnormal gene activation. C) H2Aub loops are disrupted differentially in *bmi1a/b/c* and *ring1a/b* mutants. In *bmi1a/b/c* mutants, loop disappearance is more dramatic, and new anchors become involved in forming abnormal chromatin loops. In *ring1a/b* mutants, original anchors are more likely to change their interaction partners, leading to the formation of new abnormal chromatin loops.

PcG complexes are essential in regulating gene expression in eukaryotes by depositing histone modifications and organizing chromatin to provide an appropriate chromatin environment. In mammals and *Drosophila*, PRC1 and PRC2 have close relationship in gene repression, showing extensive co‐localization of H3K27me3 and H2Aub throughout the genome.^[^ ^1^ ^]^ Discussions on PcG‐mediated repression often ignore the contribution of H2Aub. In *Arabidopsis*, a considerable number of genomic regions are marked exclusively by H2Aub.^[^ ^8a^ ^]^ This raises important questions about the function of PRC1/H2Aub and whether these only‐H2Aub regions remain spatially independent of H3K27me3. Our C‐Hi‐C data reveal the presence of PRC1‐mediated H2Aub loops in the *Arabidopsis* genome. Moreover, H2Aub‐enriched regions preferentially interact with regions enriched with the same histone modification rather than regions without H2Aub enrichment. Except for H2Aub, other histone modifications, including H3K27me3,^[^ ^13d^ ^]^ H3K4me3,^[^ ^13^, ^20^ ^]^ H3K9ac‐enriched regions preferentially interact with themselves.^[^ ^21^ ^]^ This suggests that regions enriched by identical histone modifications may cluster and be co‐regulated. We further found that although only‐H2Aub regions exist in the *Arabidopsis* genome, the majority of these regions are spatially connected to H3K27me3‐marked regions, forming PcG hub (**Figure** **6**A). Maintaining an accurate chromatin loop configuration requires an appropriate level of histone modifications (Figure 6B).

Previous study compared the gene expression levels across three types of PcG target genes, showing that genes located at only‐H2Aub regions are permissive, those in H2Aub/H3K27me3 regions are responsive, and those in only‐H3K27me3 regions are less responsive.^[^ ^8b^ ^]^ Our data support and extend this conclusion: we found that H2Aub loop formation contributes to gene repression. In addition, H3K27me3 histone modifications have a more dominant role than loop formation in gene repression. Nonetheless, loop formation further increase repression under the same histone modification conditions. Gene expression changes in *bmi1a/b/c* and *ring1a/b* mutants confirm the synergistic inhibitory effect of H2Aub loops and histone modifications at loop anchors on gene expression (Figure 6B). Moreover, we discovered that the greater the spatial interaction of H2Aub‐enriched regions with H3K27me3, the stronger the repression, providing a new perspective on PcG‐mediated repression. In *Drosophila* EDs and mouse NPCs, PRC1‐dependent chromatin loops associated exclusively with PRC1/H2Aub also exist. However, these loops are involved in developmental gene activation,^[^ ^12^ ^]^ indicating the existence of diverse mechanisms in different species. We cannot exclude the possibility that the difference is caused by experimental material variations, as our study examined whole seedlings rather than a specific tissue or a cell type. Future research should explore H2Aub loop function within dynamic processes or across different cell types. Other studies have shown that, regardless of whether chromatin structure is associated with activation or repression, it provides a platform for gene co‐regulation.^[^ ^22^ ^]^ Taken together, we propose that in *Arabidopsis*, loop formation further subdivides PcG target regions and may contribute to more precise and flexible transcriptional regulation.

TFs play an important role in regulating dynamic transcriptional responses by modulating the 3D reconfiguration of promoter‐enhancer interactions.^[^ ^21a^ ^]^ In our study, motif enrichment analysis showed that TF binding patterns distinguished Bait anchors from Nonloop Bait regions, with the latter preferentially bound by GATA TFs. GATA1 has been reported to modulate the chromatin structure, which influence the activity of the chicken α‐Globin 3′ enhancer.^[^ ^23^ ^]^ This suggests that the associated TFs, including GATAs, may be required to establish an appropriate 3D chromatin environment.

In mammals, BMI1s increase RING1s function, and both proteins reside within the same complex.^[^ ^24^ ^]^ However, in *Arabidopsis*, the situation differs to some extent. On the one hand, BMI1s have evolved ubiquitinase activity.^[^ ^5^ ^]^ On the other hand, the loss of BMI1s and RING1s results in distinct developmental deficiencies in plants.^[^ ^5^, ^25^ ^]^ Based on our molecular analyses of changes in *bmi1a/b/c* and *ring1a/b* mutants, we propose that BMI1s and RING1s have redundant functions but also individual roles. In the formation of most PRC1‐mediated H2Aub loops, they function cooperatively. Their individual roles are evident in two key aspects. First, BMI1s show a slight preference for regulating H2Aub/H3K27me3 regions and H3K27me3‐involved H2Aub loops, whereas RING1s are associated more with only‐H2Aub regions and only‐H2Aub‐loops. Second, BMI1s and RING1s play distinct roles in preventing the formation of aberrant chromatin interactions. Moreover, the overall chromatin loop configuration is differentially disrupted in the two mutants (Figure 6C). To explain the molecular basis of their distinct effects, we propose that BMI1 and RING1 proteins may each act on a subset of independent targets. Different vPRC1 complexes are present in mammalian cells, each responsible for distinct H2A monoubiquitylation pools, but work synergistically to repress Polycomb target genes.^[^ ^26^ ^]^ In *Arabidopsis*, it remains unclear whether BMI1s and RING1s function individually while exerting synergistic regulation, similar to vPRC1 complexes in mammals. In summary, our study systematically characterized the formation and function of PRC1‐mediated H2Aub loops, providing new insights for further in‐depth study in RING1s and BMI1s from the perspective of chromatin loops.

## 4 Experimental Section

### Plant Materials and Growth Conditions

The *Arabidopsis thaliana* plants used in this study were all Columbia‐0 (Col‐0) ecotypes. The mutants *bmi1a/b/c* ^[^ ^15^ ^]^ and *ring1a/b* ^[^ ^27^ ^]^ have been described previously. Mutant genotypes were identified by PCR, and the primers used were listed in Table S1 (Supporting Information).

*Arabidopsis thaliana* seeds were sterilized using a 10% sodium hypochlorite (NaClO) solution, then stratified at 4 °C in the dark for two days to promote germination. *Arabidopsis thaliana* plants were cultivated on Murashige and Skoog (MS) medium supplemented with 1% sucrose and 0.7% agar in a Percival growth chamber for ≈10 days before being transferred to soil in a greenhouse. Both growth periods were under long‐day conditions (16 h light / 8 h dark) at 22 °C. For Capture Hi‐C experiments, 10‐day‐old seedlings were harvested.

### ChIP‐Seq Data Analysis

ChIP‐Seq reads were mapped to the TAIR10 reference genome using Bowtie2.^[^ ^28^ ^]^ The resulting alignments were sorted, indexed, and compressed using SAMtools.^[^ ^29^ ^]^ Redundant reads were removed using Picard tools (v2.60; http://broadinstitute.github.io/picard/). Peak calling was performed using Sicer with ‐s tair10 ‐rt 1 ‐w 200 ‐f 150 ‐egf 0.9 ‐g 200 ‐fdr 0.05.^[^ ^30^ ^]^ Differential histone modification sites were identified using DiffBind with a default false discovery rate (FDR) ≤ 0.05, leading to an absolute log_2_ fold change being > 0.2.^[^ ^31^ ^]^ Histone modification enrichment analysis was conducted using published ChIP‐Seq data (Table S2, Supporting Information). The mean occupancy of each modification in targeted and random regions was determined using Bedmap with the ‐wmean parameter.^[^ ^32^ ^]^ Enrichment was calculated as log_2_ (target/random), and statistical significance was assessed using pairwise Mann–Whitney *U* tests. multiBigwigSummary in deepTools was used to quantify histone modification levels and to calculate mutant/wild‐type (WT) change ratios at specific anchor regions.^[^ ^33^ ^]^

### Probe Design

According to the H2Aub ChIP‐Seq results, the conserved H2Aub‐enriched regions in all WT biological duplications were identified. Probes were designed to be ≈100 bp in length. For H2Aub‐enriched regions shorter than 300 bp, a single probe‐binding site was selected at the center of the region. For regions between 300 and 500 bp in length, two probe‐binding sites were selected at one‐third and two‐thirds of the region, respectively. For regions longer than 500 bp, three probe‐binding sites were selected at one‐quarter, one‐half, and three‐quarters of the region, respectively. RNA probes were synthesized based on complementary base pairing to these probe‐binding sites (Table S4, Supporting Information). To detect the distribution of probes on the genes, bedtools were used to summarize the number of probes involved in a single gene.^[^ ^34^ ^]^

### Capture Hi‐C and Library Preparation

In situ Hi‐C was performed as previously described.^[^ ^35^ ^]^ Briefly, ≈2 g 10‐day‐old seedlings were collected for each biological replicate, with two biological replicates prepared per sample. The seedlings were fixed in 1% formaldehyde in MC buffer (10 mM KH_2_PO_4_ pH 7.0, 50 mM NaCl, 0.1 M sucrose) under vacuum at room temperature, twice for 10 min each. The fixation reaction was quenched by applying a 5‐min vacuum treatment with 0.1 M glycine. The processed plant material was homogenized in liquid nitrogen for ≈20 min before being resuspended in nuclei isolation buffer (20 mM HEPES pH 8.0, 250 mM sucrose, 1 mM MgCl_2_, 5 mM KCl, 40% glycerol, 0.25% Triton X‐100, 0.1 mM PMSF, 0.1% ß‐mercaptoethanol, 0.1% protease inhibitor cocktail). The suspension was filtered through two layers of Miracloth (Merck Millipore). The isolated nuclei were washed twice using nuclei isolation buffer, and 10–20 million purified nuclei were collected for further processing. The nuclei were then resuspended in 0.5% SDS, denatured at 62 °C for 5 min, and treated with 10% Triton X‐100 for 15 min. For chromatin digestion, 50 U *Dpn*II was added to each sample, and digestion was carried out at 37 °C overnight. The next day, the digested DNA was treated with Klenow Fragment (Thermo Scientific, EP0052) and biotin‐14‐dCTP (Invitrogen, 19518‐018) before blunt‐end repair. Chromatin ligation was performed using T4 DNA ligase (Thermo Scientific, EL0011) before adding the SDS lysis buffer (50 mM Tris‐HCl pH = 8.0, 1% SDS, 10 mM EDTA) to crush the nuclei. After decrosslinking overnight, the DNA was then isolated by phenol‐chloroform extraction, followed by ethanol precipitation. The extracted DNA was sheared by sonication using a Bioruptor Pico (Diagenode) under the following conditions: 30 s ON, 90 s OFF, at low intensity. The resulting sonicated DNA was 200–600 bp in size. VAHTS DNA Clean Beads (Vazyme, N411‐01) were used to size‐select the sheared DNA (200–600 bp), and Dynabeads MyOneTM Streptavidin C1 beads (Invitrogen, 65 001) were used to capture biotin‐14‐dCTP labeled DNA. Library preparation was performed using the VAHTS Universal Pro DNA Library Prep Kit for MGI (Vazyme, NDM608). Briefly, after biotin enrichment, on‐bead end‐repair and adapter ligation were conducted. After washing, the beads were resuspended in 10 mM Tris‐HCl buffer (pH 8.0) and incubated at 98 °C for 10 min to release the DNA from the beads. The library molecules were amplified using 12 cycles of PCR, and the products were purified using VAHTS DNA Clean Beads and eluted in ddH_2_O. For Capture Hi‐C, 1 µg of the in situ Hi‐C library was used for each experiment.^[^ ^20^ ^]^ Briefly, QuarHyb One Reagent Kit Box 2 (Dynegene Technologies) was used to hybridize the Hi‐C libraries with biotinylated RNA baits (Dynegene Technologies). QuarAcces Hyper Enrichment beads were used to enrich and separate the hybridized products, followed by amplification using QuarHyb One Reagent Kit Box 1 (Dynegene Technologies). VAHTS DNA Clean Beads were used to purify the PCR products, and the Capture Hi‐C libraries were sequenced on an MGI DNBSEQ T7 PE150 platform, generating 2 × 150 bp paired‐end reads.

### Capture Hi‐C Data Analysis

The two replication datasets were filtered and mapped to TAIR 10 using fastp^[^ ^36^ ^]^ and Bowtie2,^[^ ^28^ ^]^ respectively. Valid pairs were identified and deduplicated using HiCUP‐0.9.^[^ ^37^ ^]^ The reproducibility was measured in hicCorrelate with a range from 10 kb to 1 Mb in HiCExplorer.^[^ ^38^ ^]^ Based on Capture Hi‐C data, CHiCAGO was used to define the H2Aub loop (Table S3, Supporting Information).^[^ ^39^ ^]^ Considering the features of the *Arabidopsis* genome, the settings in CHiCAGO were adjusted as in previous research^[^ ^20^ ^]^ with the following parameters: weightAlpha = 13.5319239, weightBeta = ‐1.3100426, weightGamma = ‐10.3516115, and weightDelta = 0.1635212. The probe sites (Table S4, Supporting Information) and the *Dpn* II‐digested genome fragments were used to generate baited restriction fragments. The two replication datasets were simultaneously inputted into CHiCAGO. To evaluate the loops captured on a single gene, the maximum distance between other same‐direction anchors was defined as the span of the loops. The more concentrated the distribution of anchors, the smaller the span. For Bait regions and loop classification, baited restriction fragments overlapping with H3K27me3‐marked regions were classified as H2Aub/H3K27me3 Bait regions and loops led by these Bait regions were defined as H3K27me3‐involved‐loops. Loop anchor annotation was performed using ChIPpeakAnno^[^ ^40^ ^]^ and GenomicFeatures^[^ ^41^ ^]^ with 1000 bp upstream regions defined as promoter regions. For motif enrichment analysis, findMotifsGenome.pl in HOMER was used with the ‐size 50 parameters.^[^ ^42^ ^]^

### RNA‐Seq Data Analysis

For the RNA‐Seq data, sequencing reads were mapped to the reference genome using HISAT2.^[^ ^43^ ^]^ The results were sorted, indexed, and compressed, following the same approach as in ChIP‐Seq data analysis. StringTie was used for transcription quantification.^[^ ^44^ ^]^ Differentially expressed genes were identified using DEseq2, with an adjusted *P*‐value (*p*_adj_) < 0.05 and |log_2_FoldChange| ≥ 1.^[^ ^45^ ^]^ Gene ontology (GO) analysis was performed using the clusterProfiler R package.^[^ ^46^ ^]^

### 3C‐qPCR

Chromosome Conformation Capture (3C) experiments were performed according to the in situ Hi‐C protocol described above, with several modifications. The experimental steps up to *Dpn*II digestion on the first day were identical to those used in the Hi‐C protocol. The next day, the digested chromatin was directly ligated using T4 DNA ligase (Thermo Scientific, EL0011) prior to the addition of SDS lysis buffer (50 mM Tris‐HCl pH 8.0, 1% SDS, 10 mM EDTA) to crush the nuclei. After overnight decross‐linking, the DNA was isolated by phenol‐chloroform extraction followed by ethanol precipitation. The purified DNA was dissolved in 400 µL H_2_O. 3C DNA ligation products were quantified by real‐time quantitative PCR (qPCR) using the ChamQ Universal SYBR qPCR Master Mix (Vazyme, Q711‐02) on an Agilent AriaMx Real‐Time PCR System. Data are scaled to set the highest interaction point per fixed primer to 1 and normalized by the interaction frequency of the 3C anchor fragment and the negative region between WT and mutants.^[^ ^47^ ^]^

### RNA Extraction, Reverse Transcription and RT‐qPCR

Total RNA was extracted from 10‐day‐old seedlings grown under long‐day conditions using the E.Z.N.A. Plant RNA Kit (Omega, R6827‐01). Approximately 0.1 g of seedlings was used per biological replicate, with three biological replicates collected for each sample. RNA (1 µg) was used to synthesize complementary DNA (cDNA) for RT‐qPCR (quantitative reverse‐transcription PCR) using the EasyScript One‐Step gDNA Removal and cDNA Synthesis SuperMix Kit (TransGen, AE311). RT‐qPCR was performed using the ChamQ Universal SYBR qPCR Master Mix (Vazyme, Q711‐02) on an Agilent AriaMx Real‐Time PCR System.

## Conflict of Interest

The authors declare no conflict of interest.

## Author Contributions

L.L., M.Y., Z.S., Y.L. contributed equally to this work. L.L., Z.S., Y.L., S.X. performed all the experiments. M.Y., D.W. and H.H. analyzed high‐throughput sequencing data. L.L., M.Y. and Y.Z. interpreted the data. L.L., M.Y., M.C. and Y.Z. planned the experiments and wrote the manuscript. All authors read and approved the final manuscript.

## Supporting information



Supporting Information

Supplemental Table 1

Supplemental Table 2

Supplemental Table 3

Supplemental Table 4

## Data Availability

The capture Hi‐C raw sequence data reported in this paper have been deposited in the Genome Sequence Archive in National Genomics Data Center, China National Center for Bioinformation / Beijing Institute of Genomics, Chinese Academy of Sciences (GSA: CRA023402) that are publicly accessible at ^https://ngdc.cncb.ac.cn/gsa.[48]^ RNA‐Seq and ChIP‐Seq data can be found in the European Nucleotide Archive (ENA) under the accession number PRJEB77931.
