# Supplementary material for: PRC1‐Mediated H2Aub Loop Formation and Function in Arabidopsis
Source: Adv Sci (Weinh). 2025 Jul 12;12(35):e04377. doi: 10.1002/advs.202504377 (PMC12463081; doi:10.1002/advs.202504377)
Supplement: Supplementary file 1 — Supporting Information [file ADVS-12-e04377-s002.docx]

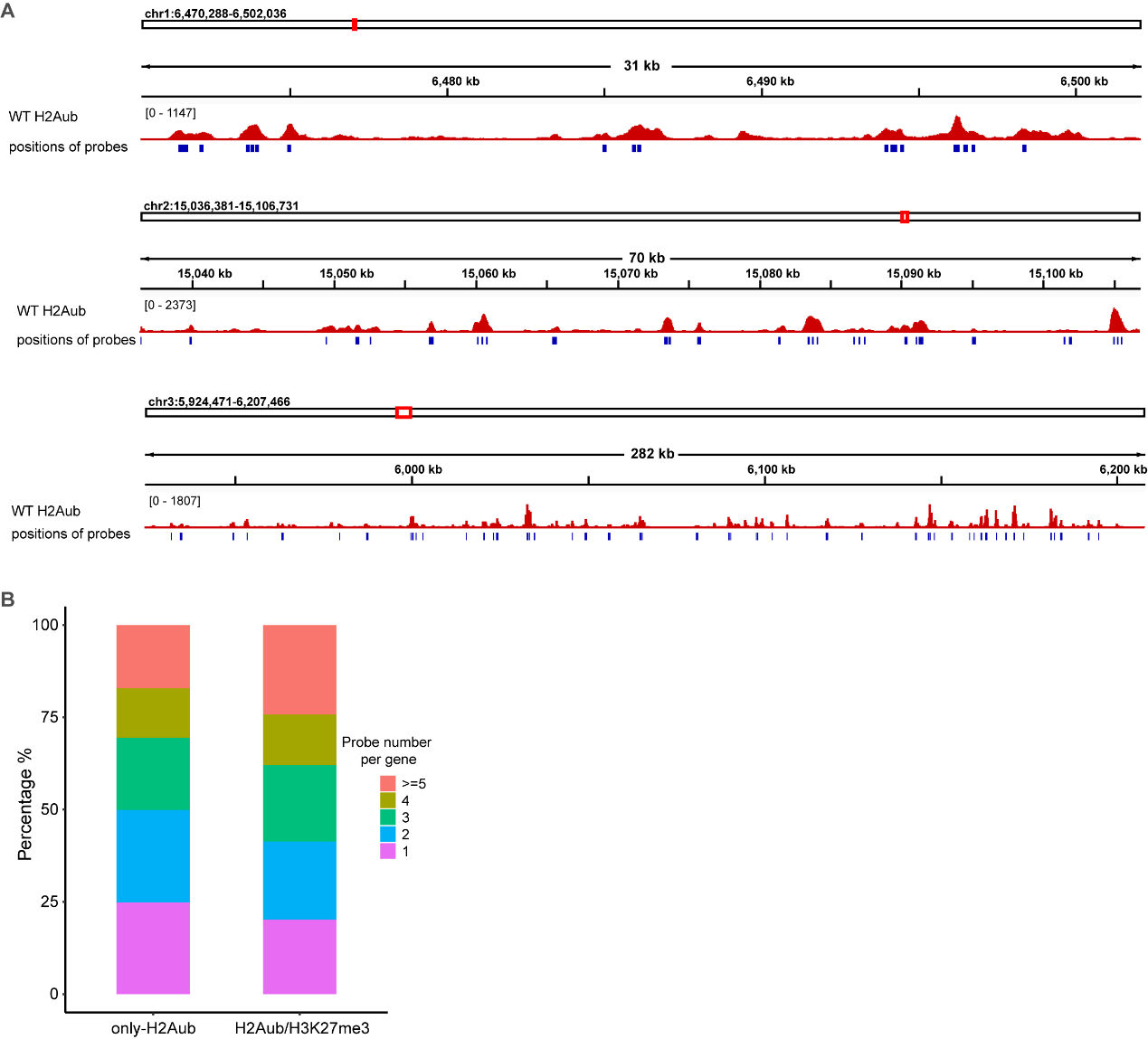


**Figure S1.** **The designation and distribution characteristics of H2Aub probes.** **A)** Genome browser images showing the positional information of probes used in this study. Three different scales are displayed. The H2Aub ChIP-Seq signals are showed. The positions of probes are indicated by the blue boxes at the bottom. **B)** Number of probes per gene. Bar plots show the percentage of genes with different numbers of probes in only-H2Aub-modified genes (left) and H2Aub/H3K27me3-modified genes (right).


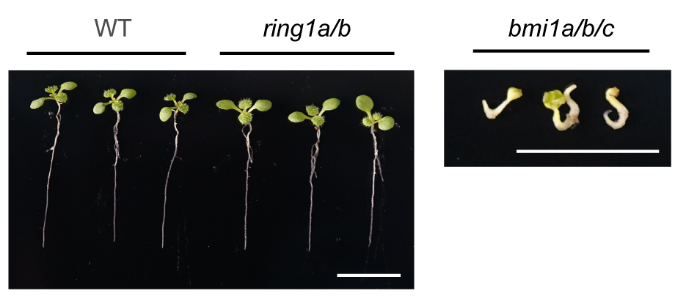


**Figure S2.** **The plant materials involved in this study.** Phenotypes of 10-day-old seedlings of WT plant, *ring1a/b* and *bmi1a/b/c* mutants (from left to right). Scale bar: 1 cm.


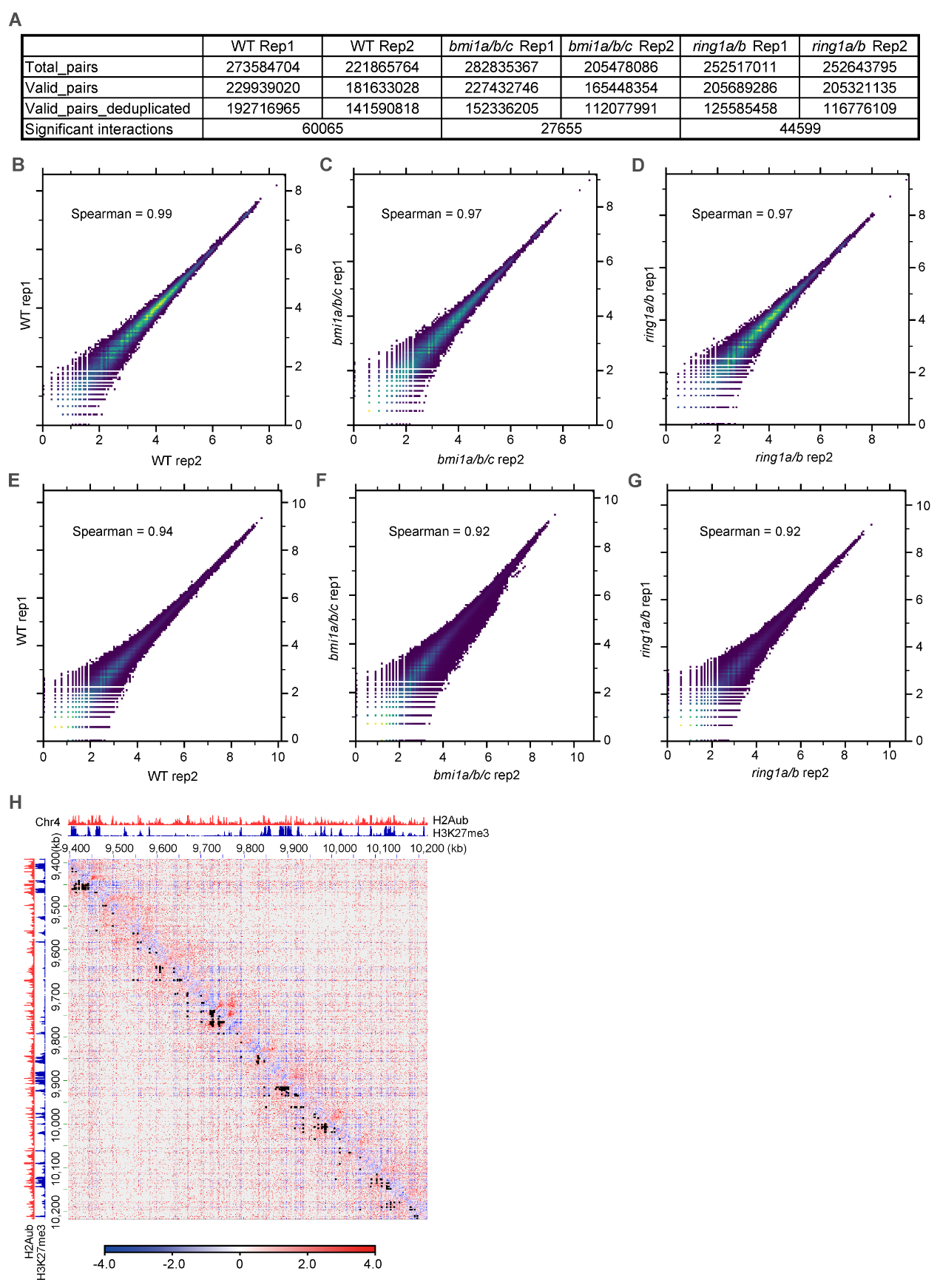


**Figure S3.** **Overview of H2Aub Capture-Hi-C for WT, *bmi1a/b/c* and *ring1a/b*.** **A)** Table summarizes the pair and interaction information of the Capture-Hi-C data. **B–D)** Repeatability test of Hi-C in WT (B), *bmi1a/b/c* (C), and *ring1a/b* (D), assessed using Spearman’s rank correlation. **E–G)** Repeatability test of C-Hi-C in WT (E), *bmi1a/b/c* (F) and *ring1a/b* (G), which was applied by Spearman correlation. **H)** Contact heatmaps of WT normalized by Observed/Expected, showing the H2Aub loops indicated by black marks in the bottom left. A region in chromosome 4 is presented as the example. H2Aub and H3K27me3 ChIP-Seq signals are displayed at the left and top of the heatmaps.

**
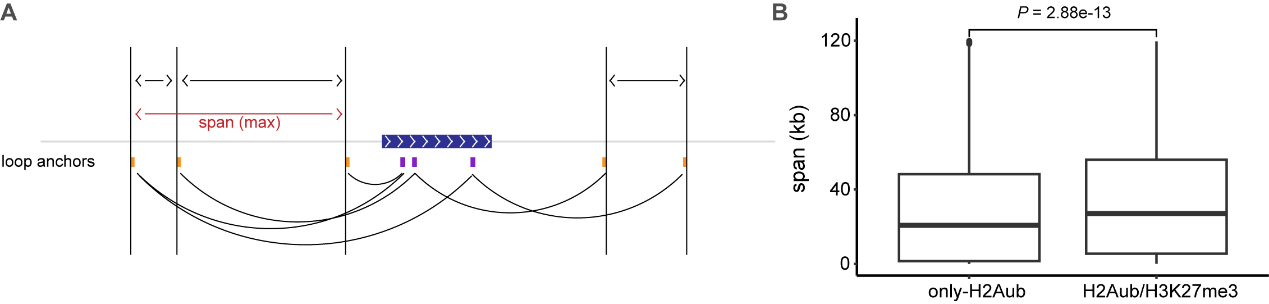
Figure S4. The variation degree of H2Aub loops from a single gene. A)** Schematic diagram illustrating the method used to characterize the degree of variation of different loops from a single gene. The blue box indicates a single gene, with its direction shown by a white arrowhead. Boxes below the gene schematic represent loop anchors. Purple boxes denote anchors located within a single gene, while orange boxes represent the other anchors of loops from the same gene. The distance between the two other anchors from a single gene is evaluated from the same side of the gene. The span of anchor interacting with one gene is indicated by the red line segment with arrows at both ends, which is the maximum distance between the two other anchors of the single gene. **B)** The variation degree of loops for individual genes. The variation degree is represented by anchor span values for only-H2Aub-modified genes (left) and H2Aub/H3K27me3-modified genes (right). *P*-values were calculated using the Wilcoxon rank-sum test.

**
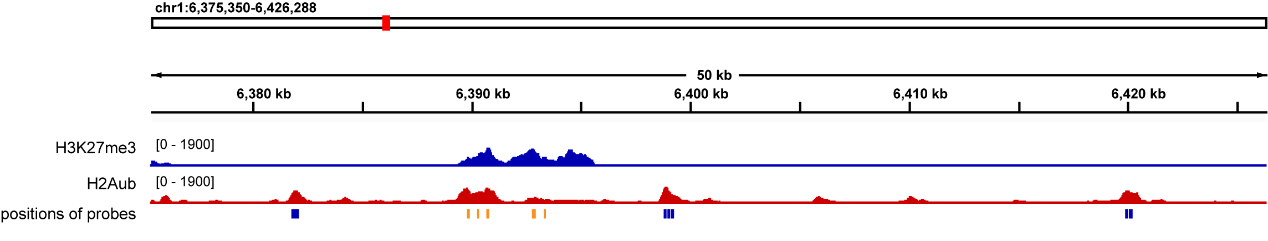
**

**Figure S5. The classification of H2Aub bait regions.** H3K27me3 and H2Aub ChIP-Seq results are displayed, and the baits are classified by whether they have H3K27me3 enrichment. Only-H2Aub and H2Aub/H3K27me3 bait regions are indicated by blue and orange boxes in the bottom track, respectively.


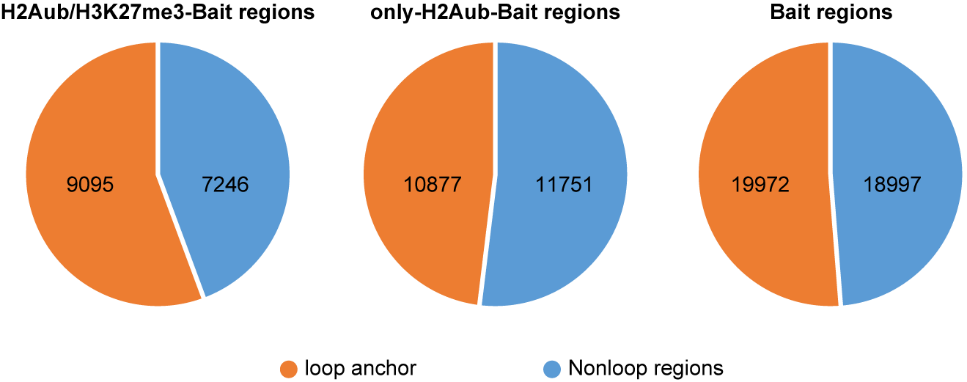


**Figure S6.** **Proportions of loop-forming and nonloop bait regions in H2Aub/H3K27me3, only-H2Aub, and all Bait regions.** Pie charts showing the proportion of the Bait regions that form loops (loop anchor) or do not form loops (nonloop regions). The distributions for H2Aub/H3K27me3-Bait regions, only-H2Aub-Bait regions, all Bait regions are shown from left to right.


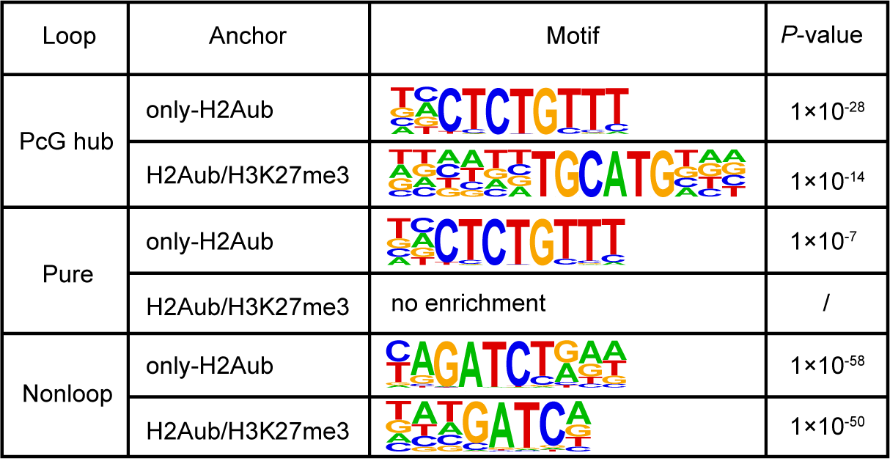


**Figure S7.** **The first ranked motif enriched at the six types of loop anchors.** The anchors were classified based on the histone modification they had and the loop they involved. The enriched motif and corresponding significance are indicated.

**
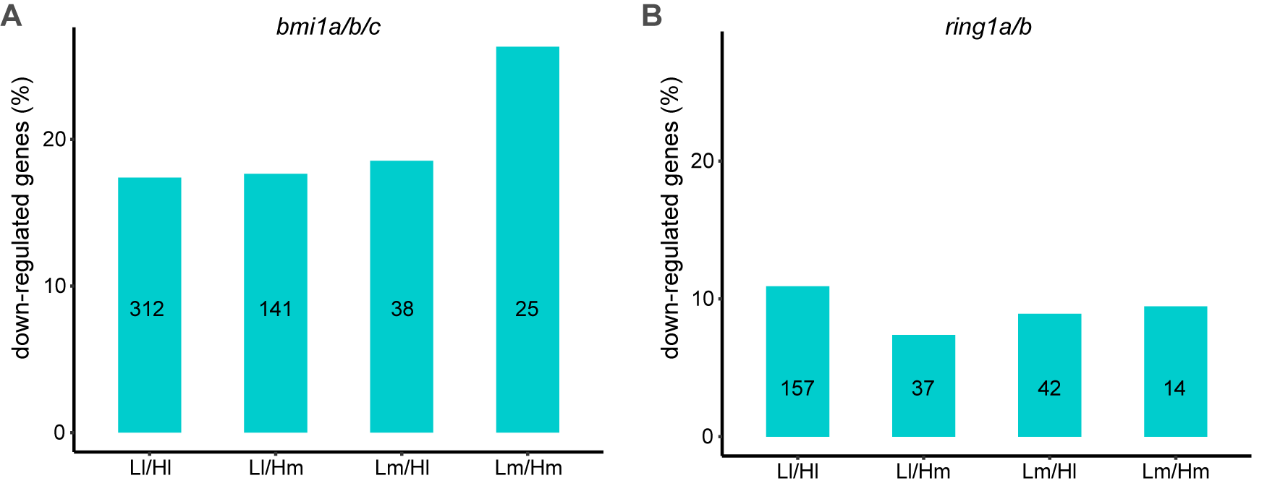
**

**Figure S8.** **Down-regulated genes at anchors associated with four types of loops in the two PRC1 mutants.** Bar plots showing the percentage of down-regulated genes in *bmi1a/b/c* (A) and *ring1a/b* mutants (B). From left to right, the results are shown for anchors corresponding to four loop types: Ll/Hl, Ll/Hm, Lm/Hl, and Lm/Hm. The number of genes in each group is shown within each bar.

**
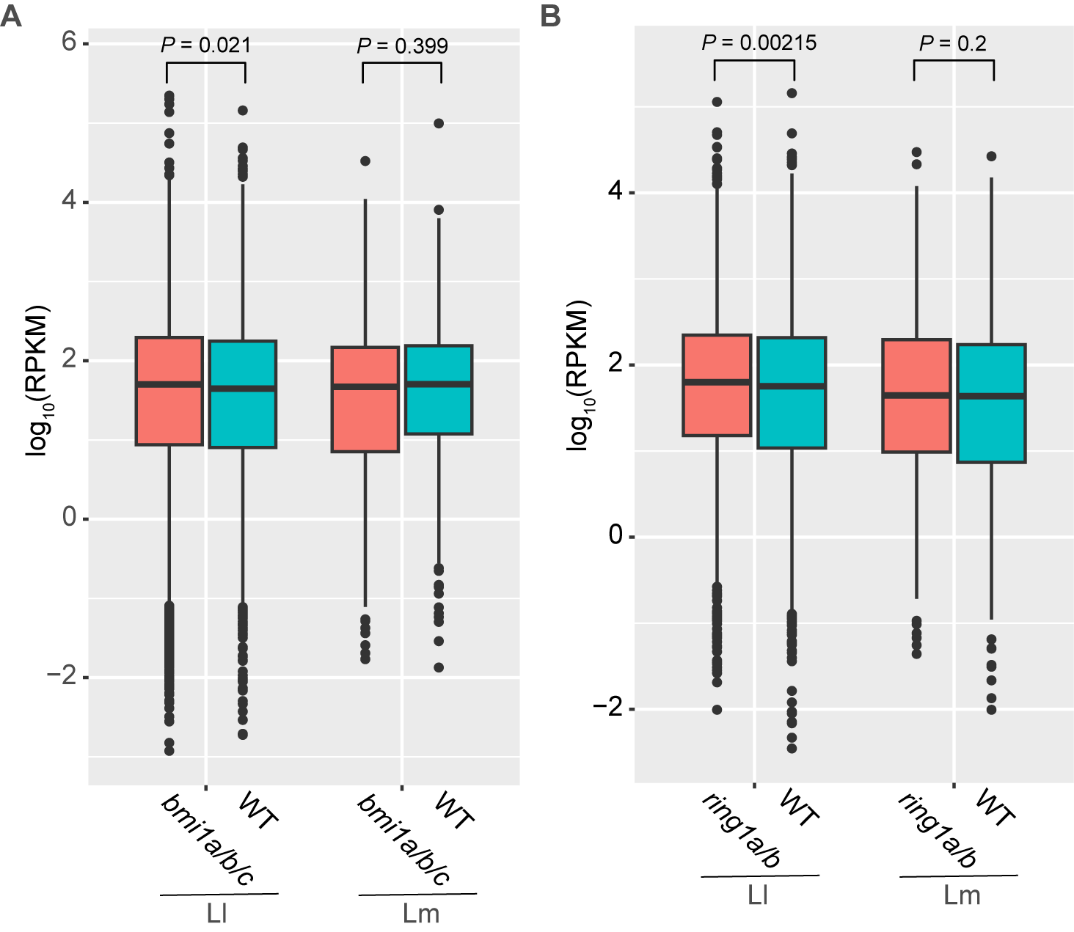
**

**Figure S9. Expression levels of genes at maintained and disappeared loop anchors in WT and two PRC1 mutants.** **A)** Box plots showing the comparison of gene expression levels between *bmi1a/b/c* mutants and WT at disappeared loop (Ll) anchors (left) and maintained loop (Lm) anchors (right). **B)** Box plots showing the comparison of gene expression levels between *ring1a/b* mutant and WT at anchors of disappeared loops (LI, left) and maintained loops (Lm, right). *P*-values were calculated using the Wilcoxon rank-sum test.

**
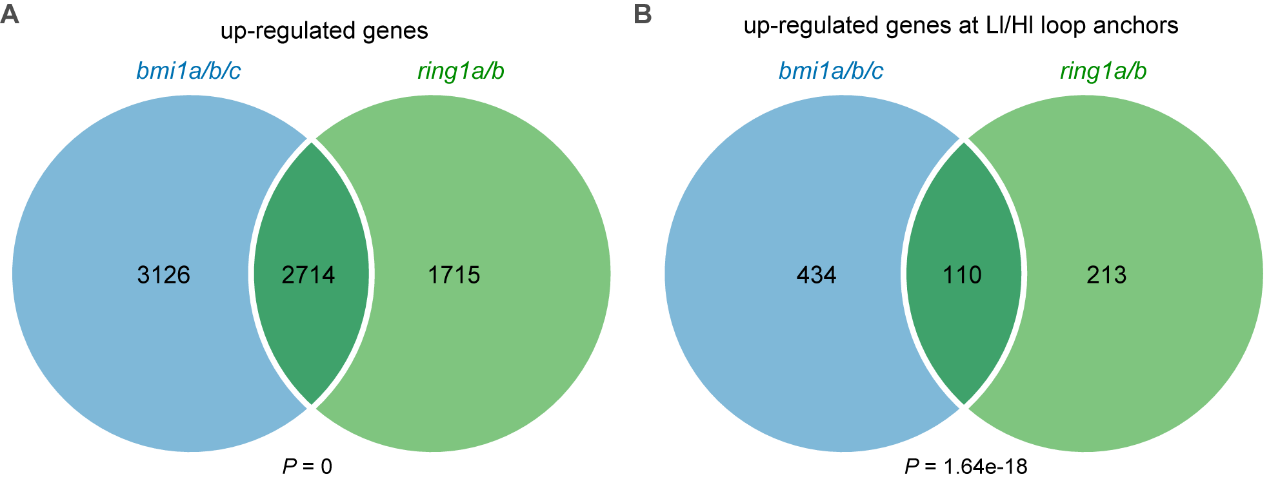
**

**Figure S10. Comparison of differentially expressed genes in *bmi1a/b/c* and *ring1a/b* mutants. A)** Venn diagram showing the overlap of all up-regulated genes in *bmi1a/b/c* and *ring1a/b* mutants. **B)** Venn diagram showing the overlap of up-regulated genes at Ll/Hl loop anchors in *bmi1a/b/c* and *ring1a/b* mutants. *P*-values were calculated using the Fisher's exact test.


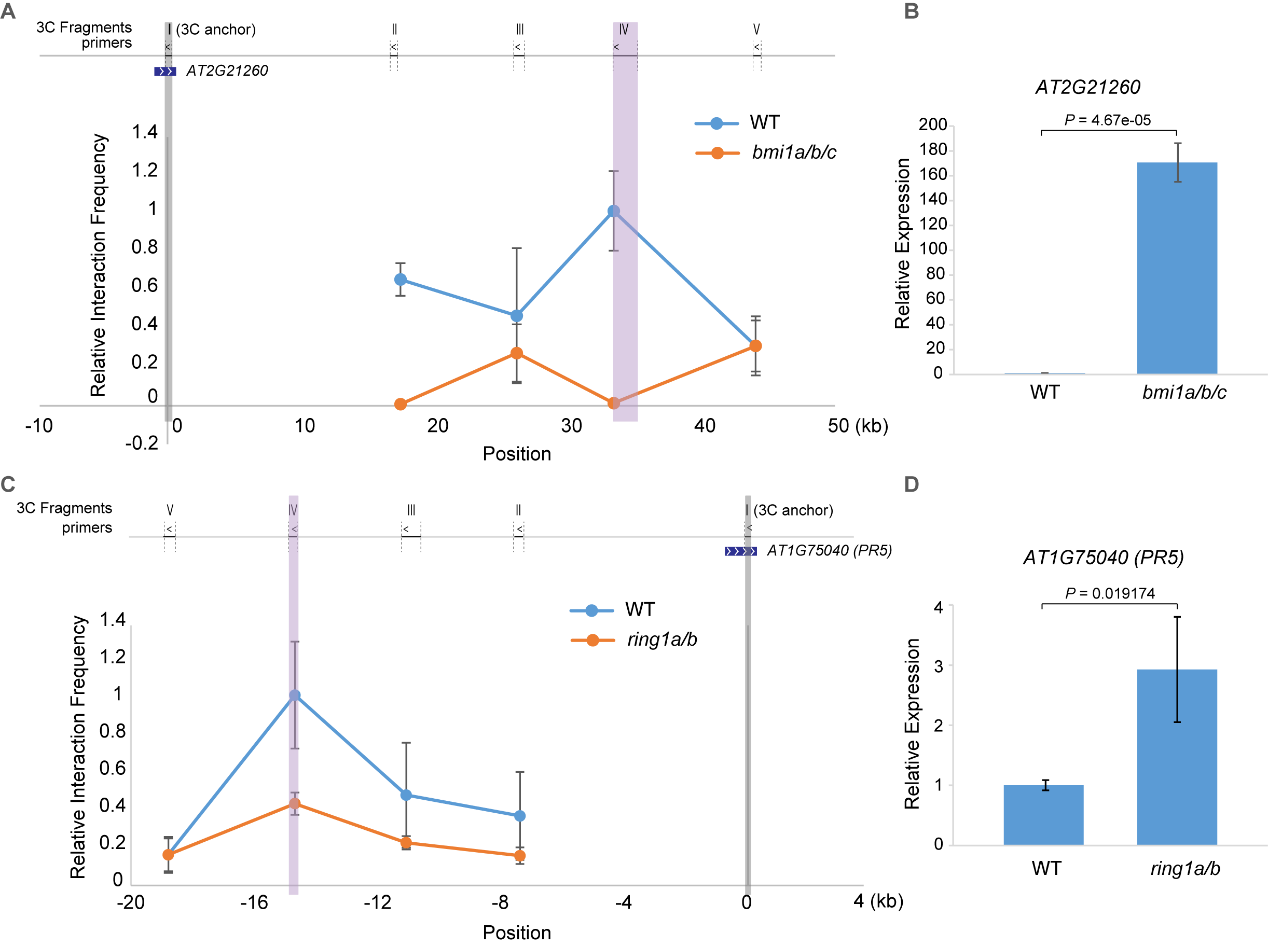


**Figure S11.** **Verification of differences in loop and expression between PRC1 mutants and WT plants. A,C)** 3C-qPCR analyses show that the H2Aub loop in WT plants in the example is disrupted in *bmi1a/b/c* (A) or *ring1a/b* (C) mutants. The top scale diagrams indicate the positions of up-regulated genes at Ll/Hl loop anchors in *bmi1a/b/c* (A) or *ring1a/b* (C) mutants. All tested *Dpn* II fragments are shown as black lines and are labeled with Roman numerals; dashed lines indicate restriction sites. Primers were designed on five restriction fragments as marked by black arrowheads. The tested anchor fragment (3C anchor, fragment I) is highlighted in grey, and the corresponding anchor in WT is highlighted in purple. The fixed primer was placed on the 3C anchor fragment I and tested against all other primers to measure interactions. The example gene is represented by a blue box, with white arrows indicating transcriptional direction. The X-axis shows genomic positions (in kb) relative to the 3C anchor fragment. The Y-axis indicates relative interaction frequency. Data are scaled to set the highest interaction point per fixed primer to 1 and normalized by the interaction frequency of the 3C anchor fragment and the negative region between WT and mutants. WT and PRC1 mutant data are shown in blue and orange, respectively. Error bars represent the standard deviation from three biological replicates. **B,D)** RT-qPCR analyses show that the example gene is up-regulated in *bmi1a/b/c* (B) or *ring1a/b* (D) mutants. Relative expression levels of *AT2G21260* (*bmi1a/b/c* vs. WT) and *AT1G75040* (*PR5*) (*ring1a/b* vs. WT) are shown. *PP2A* was used as the reference gene. Error bars represent the standard deviation from three biological replicates. *P*-values were calculated by ANOVA.

**
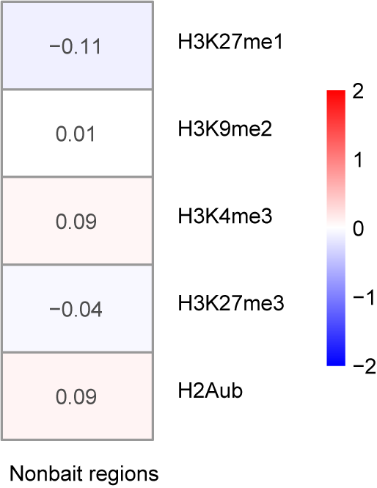
**

**Figure S12.** **The histone modification enrichment analysis at Nonbait anchors of WT.** The depletion of histone modification is shown in blue and enrichment in red (target regions vs. randomly selected regions). Published data were obtained from the Plant Chromatin State Database and are listed in Table S2 (Supporting Information).


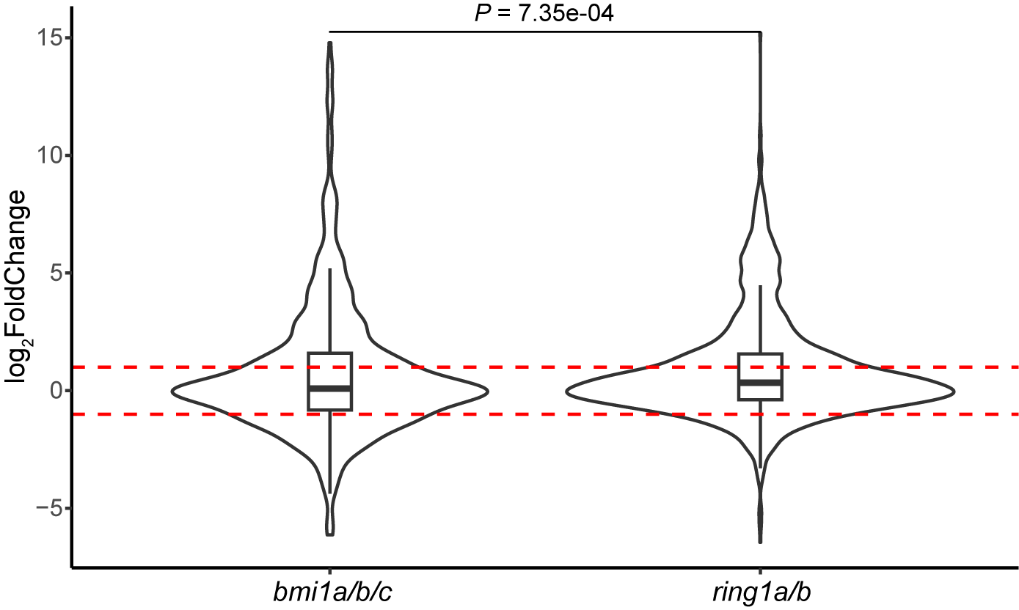


**Figure S13.** **Changes in gene expression at new anchors in *bmi1a/b/c* and *ring1a/b* mutants.** Violin plots showing the distribution of genes at new anchors (anchors belonging only to newly formed loops) in *bmi1a/b/c* (left) and *ring1a/b* (right) mutants, based on fold change in gene expression. Red dashed lines indicate |log_2_FoldChange| = 1. The *P*-value was calculated using the Wilcoxon rank-sum test.
